# Alternating movement strategies of a tropical raptor

**DOI:** 10.1038/s41598-025-11248-8

**Published:** 2025-08-13

**Authors:** Eben H. Paxton, Kristina L. Paxton

**Affiliations:** 1 Pacific Island Ecosystems Research Center, U.S. Geological Survey, Box 44, Hawai‘i National Park, HI 96718 USA; 2 Hawai‘i Cooperative Studies Unit, University of Hawai‘i Hilo, PO Box 44, Hawai‘i National Park, , HI 96718 USA; 3 Present Address: Natural Resource Management, Hawai’i Volcanoes National Park, PO Box 52, Hawai’i National Park, HI 96718 USA

**Keywords:** Ecology, Conservation biology

## Abstract

The majority of raptor species reside in the tropics, yet very little is known about their movement ecology. However, quantifying movement behavior can provide otherwise elusive information on resource needs, habitat selection, and ecological constraints, which is important for understanding ecological patterns and the management of species of conservation concern. On the Island of Hawai‘i, Hawai‘i, USA, the endemic ‘Io, or Hawaiian Hawk (*Buteo solitarius*), is a species of conservation concern that little is known of their movement ecology, yet they are dependent on a fragmented and rapidly changing environment. We tracked 15 individuals for up to 18 months across a diverse landscape on the eastern side of the island. We found that all ‘Io occupied a relatively small geographic area, their place of residency, where they spent all or most of their time. However, 10 individuals also exhibited an alternative movement pattern, where individuals repeatedly commuted back and forth between their place of residency to another, geographically disjunct location. These commuter periods, which could last from 24–180 days, were characterized by frequent (9–259) movements, with individual trips lasting 4–77 h away from their place of residency and 12–47 h in between commuter trips. In most cases, individuals went to the same non-contiguous commuting destination, even across multiple commuting sessions, indicating high fidelity to commuting locations. The ‘Io is a forest adapted Buteo but occurs across a diverse landscape from forest to agriculture lands to urban areas. Habitat selection analysis indicated high individual variation among different birds, but generally a preference for forest patches at localized levels. The discovery of the alternative commuting strategy for many ‘Io represents a cryptic movement pattern in the species, demonstrating the power of small, long-lived Global Position System tracking devices to track movement and providing important insights into the ecology of a tropical island raptor.

## Introduction

Animals move to acquire critical resources such as food, mates, and shelter, avoid predators and competitors, and respond to changes in their environment. Patterns of movement behavior are therefore shaped by a species’ ecology, community, and habitat^1^. Tropical birds are generally considered sedentary^2^, but many Neotropical raptors show movement patterns from migratory to irregular localized movements^3^, and recent tracking studies have revealed that a number of species once considered sedentary conduct varying levels of movement^2^. The tropics encompass a wide range of habitats and climates, and variability in climate and seasons can have strong effects on resource availability^4,5^, which could affect movement behaviors. However, tropical raptors, while representing the greatest diversity of raptors globally, have been the subject of relatively little scientific research^6^. Given the cognitive linkage between an animal’s movement and how it perceives its environment^1^, quantifying movement can provide otherwise elusive information on resource needs, habitat selection, and ecological constraints, which is important for understanding ecological patterns and the management of species of conservation concern^7^.

The ‘Io, or Hawaiian Hawk (*Buteo solitarius*) is an endemic raptor on the Island of Hawai‘i, Hawai‘i, USA. The Hawaiian Islands are aseasonal but have strong climatic clines both in elevation (mountains up to 4,205 m above sea level) and precipitation gradients from east (wet) to west (dry) as easternly trade winds interact with the tall mountains to create orthogonal rainfall patterns^8^. Little is known about ‘Io movement behavior^9^, but they are believed to be year-round residents that maintain territories with a breeding period from early April through August^10^. High endemism and low population size led to the raptor being listed under the U.S. Endangered Species Act as an endangered species in 1967. However, multiple surveys of ‘Io over time indicated stable populations widely distributed across the island^9,11,12^, which led to the ‘Io being delisted in 2020^13^. Nonetheless, they are still a species of conservation concern given their low estimated global population size of 3,239^11^ and endemism to a single island. ‘Io are considered a forest raptor^10^, but today they are detected across a wide range of habitats^11^, many that are highly modified from the structure of the forest habitat that once covered the islands^14^.

The fragmentation and modification of many forested areas of Hawai‘i created novel habitat that likely altered resource distribution, which should result in altered hunting behavior and movement behavior. Habitat loss and fragmentation is a major threat to tropical raptors worldwide^6^, both reducing abundance and distribution^15^ as well as potentially changing hunting behavior and efficiency^16,17^. If ‘Io require large tracks of forest within their home ranges, then home ranges in developed landscapes may be larger than continuous forest habitats. Birds having to travel over larger areas to find sufficient resources would expend greater energy, potentially reducing fitness^18^. Alternatively, if ‘Io occupy small home ranges, as indicated from past studies^10^, then loss of suitable habitat may reduce the areas they can occupy, but that the size of the home ranges would vary by the habitat type. To address these questions, we placed tracking devices on ‘Io sampled from forested, agricultural, and developed (urban) habitats across the eastern side of the Island of Hawai‘i, and tracked them to (1) quantify their movement patterns, (2) estimate their home range sizes, and (3) evaluate habitat preferences at local and landscape scales across a gradient of urban to forested landscapes. We hypothesized that ‘Io in minimally disturbed forest would have the smallest, most contiguous home ranges, as the intact habitat would provide maximum resource density, while ‘Io in more degraded habitat would have larger home ranges necessary to encompass sufficient suitable habitat.

## Methods

### Study site and species

**Fig. 1:**
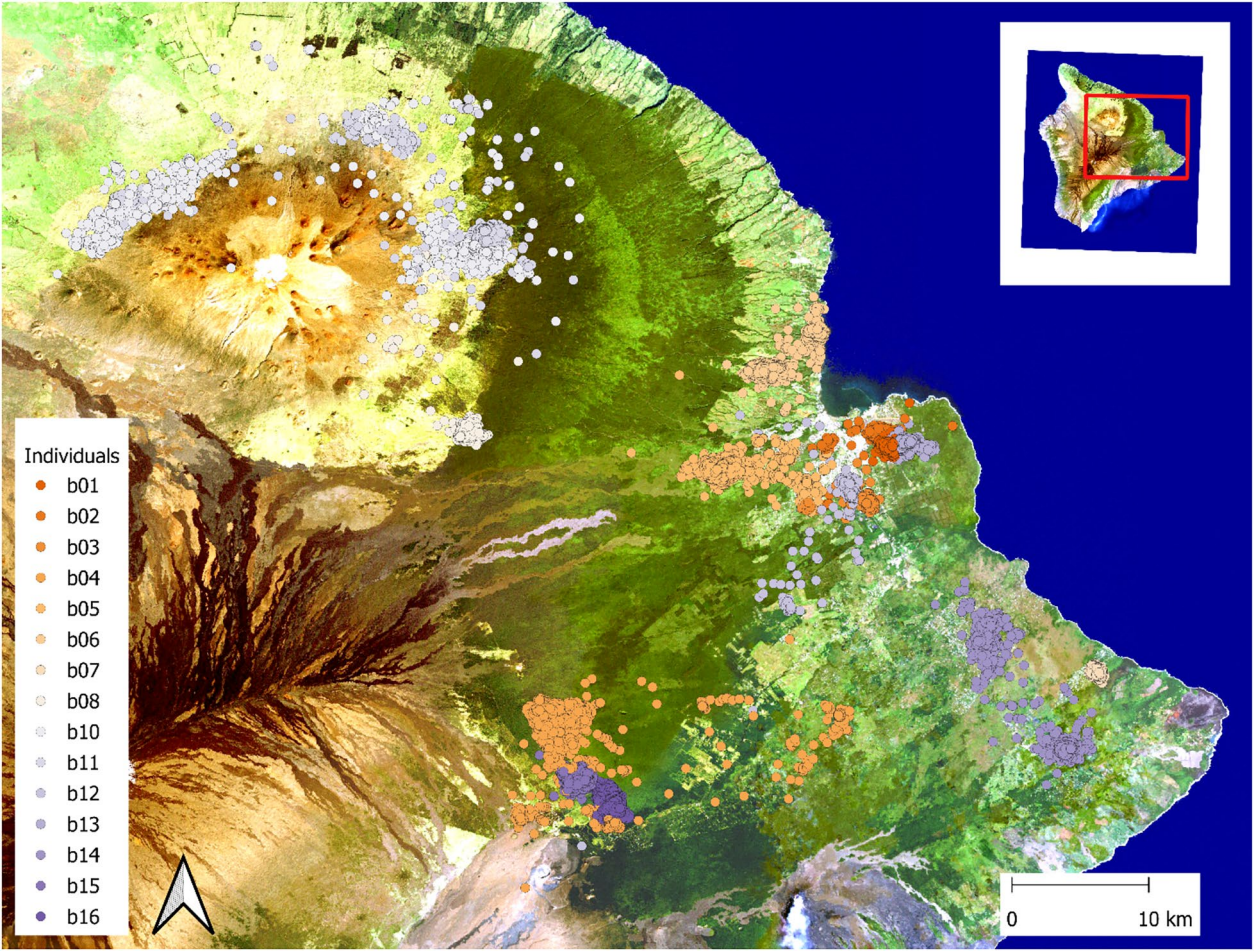
Distribution of locations detected for 15 ‘Io (*Buteo solitarius*) on the Island of Hawai‘i, 2019–2021. Image created using QGIS (v. 3.40.7, qgis.org) using publically avaialble 2002 LandSat satellite imagery for the base layer (NASA/USGS).

The ‘Io is a small (average mass 606 g female, 441 g male) Buteo hawk endemic to the Island of Hawai‘i, where it occurs from the coast to treeline (> 2,000 m elevation)^19^. Although considered a forest habitat species, the ‘Io occurs in habitats ranging from intact native forest to fragmented rural and agricultural landscapes, and is frequently observed in urban areas^19^. ‘Io for this study were sampled over a large portion of the eastern (windward) side of the Island of Hawai‘i in the Native Hawaiian land divisions (moku) of Kaʻū, Puna, and Hilo (Fig. 1). Individuals were targeted for capture in native forest habitat, rural mixed agriculture lands, and in urban or near urban habitats. Although the sampling design was intended to provide a representative sample of individuals from each habitat type for comparison, capture location was not necessarily indicative of the dominant habitat in which an individual occurred in (refer to Results).

### Field methodology

Birds were captured from December 2019 to February 2020 (Table 1) using bal-chatri traps^20^ with wild caught Black Rats (*Rattus rattus*) as lure animals. Only adults were chosen for this study, determined by morphometric characteristics^19^, and while both male and female ‘Io were captured, males are ~ 30% smaller than females and most males captured were too small to meet the 4% limit of transmitter-to-body weight ratio we used to ensure that the transmitters had minimal influence on movement behavior. We used Cellular Tracking Technology (Rio Grande, New Jersey) GPS-GSM (global positioning system location with cellular connectivity) transmitters that were attached via a backpack harness using 6.4-mm wide Electra ribbon (BallyRibbon Mills, Pennsylvania) with a cotton thread break away design to allow the harness to fall off after several years. Most GPS-GSM tags were 16 g but we trialed 2 lighter 14-g tags to track 2 males. Most tags were programmed to record 1 location per hour during the day (0600–1800) and every 3 h during the night (1800–0600) for a total of 16 locations per day. Two tags had alternative programs to record 1 location every 2 h throughout a 24-h period due to poor recharging performance. All birds were released at the capture location.

**Table 1:** Summary of movement data collected from individual ‘Io (*Buteo solitarius*) tracked with GPS-GSM transmitters on the Island of Hawai‘i, 2019–2021. Individuals were captured from four general regions, Hilo township, Puna District, Hakalau Forest National Wildlife Refuge on Mauna Kea, and Kaʻū (Volcano) township on Mauna Loa, and were captured in one of three habitat types: forest, rural mixed agriculture, and developed. Start and end dates indicate the specific days each bird was tracked for this study, with associated number of locations and days detected. Hatching dates of nests were estimated for female ‘Io based on a large increase in the time spent at the presumed nest site location. Years without a date indicate that the female did not appear to breed unless otherwise indicated.

Bird ID	Region	Capture habitat	Sex	Start date	End date	No. locations	Days detected	Hatching date 2020	Hatching date 2021
b01^a^	Hilo	Developed	Female	15-Dec-2019	30-Jul-2021	7630	538	11-Jun-2020	10-Jun-2021
b02	Hilo	Developed	Male	21-Dec-2019	3-Sep-2020	3986	258	–	–
b03	Hilo	Rural	Female	23-Dec-2019	30-Jul-2021	6561	563	16-May-2020	10-Jun-2021
b04	Kaʻū	Developed	Female	27-Dec-2019	30-Jul-2021	8011	546	–	20-Jun-2021
b05	Hilo	Rural	Female	2-Jan-2020	30-Jul-2021	8514	551	8-May-2020	–
b06	Hilo	Rural	Female	6-Jan-2020	30-Jul-2021	8959	553	15-Jun-2020	–
b07	Puna	Developed	Female	13-Jan-2020	30-Jul-2021	9162	555	5-Jun-2020	28-May-2021
b08^b^	Hakalau	Forest	Female	17-Jan-2020	3-Jun-2020	2384	139	–	–
b10^c^	Hakalau	Forest	Female	19-Jan-2020	28-Jul-2021	9523	557	3-Aug-2020	–
b11	Hakalau	Forest	Female	20-Jan-2020	30-Jul-2021	9374	548	–	23-Jul-2021
b12^a^	Hilo	Developed	Female	4-Feb-2020	30-Jul-2021	8070	510	11-Jun-2020	20-Jun-2021
b13	Hilo	Developed	Female	8-Feb-2020	30-Jul-2021	8936	535	–	5-Jun-2021
b14^a^	Puna	Forest	Female	13-Feb-2020	30-Jul-2021	4965	518	22-Jun-2020	19-Jul-2021
b15	Kaʻū	Rural	Female	15-Feb-2020	30-Jul-2021	7215	490	12-Jun-2020	16-May-2021
b16	Kaʻū	Rural	Male	15-Feb-2020	9-Jun-2021	8030	481	–	–

^a^ Presumed nesting locations differed between years.

^b^ Individual appeared to be nesting, but tag stopped transmitting in 2020 prior to presumed hatching date.

^c^ Individual appeared to be nesting, but tracking period in 2021 stopped before presumed hatching date.

We analyzed tracking data for ‘Io starting 48 h post release until the GPS-GSM tag stopped transmitting data or 31 July 2021, whichever period was longer. We removed all location estimates that were based on fewer than three satellites or two-dimensional position accuracy (n = 97), and we used the ‘outlie’ function in the R package ctmm^21^ to identify and exclude improbable locations based on a combination of flight speeds and distance moved from a bird’s core area (n = 14). The tracking data are available at Paxton and Paxton^22^. We assessed tracking location accuracy from 5 stationary GPS-GSM tags placed outside at a fixed location for 4 days (n = 122 locations) using the function ‘uere.fit’ in the R package ctmm^21,23^. The average horizontal and vertical location errors of our GPS-GSM tags were estimated to be 5.9 m (95% confidence interval [CI]: 5.4,6.4) and 6.7 m (95% CI: 5.8, 7.5), respectively.

### Behavioral segmentation

Based on visual assessment of movement data variograms for each ‘Io, which show the autocorrelation structure of movement data^24^, we concluded that most individuals lacked range residency (i.e., tendency of an animal to stay within its home range) and were likely shifting their range during the time period of tracking. Therefore, we used a segmentation method in the R package segclustd^25^to identify potential shifts in an individual’s space use across their tracking period. This method detects change points in a time series of locations (Universal Transverse Mercator [UTM] Easting and Northing coordinates) based on shifts in either the mean location or the variance of the locations. We set the minimum segment length to 225 locations (approximately 2 weeks) to avoid short-term shifts in space use. For individuals with multiple segments identified by the segmentation analysis, we classified segments into two states, stationary and commuting, based on large differences in the standard deviation of the Easting and Northing locations among segment types. Stationary states are characterized by relatively constrained movements within a localized area, while commuting states are characterized by frequent movements away from and back to the same localized areas used during stationary states. We used a linear mixed model in R (lme4 package^26^,) to quantify differences in the standard deviation of Easting and Northing locations (log transformed for normality) between segments classified into different states, with individual ID included in the model as a random variable to account for multiple segments from the same individual. We visually examined segmentation plots (Figs. 3, S3) to determine if birds exhibited site fidelity (i.e., a tendency to return to the same general area) in their stationary and commuting areas.

### Home range estimates

For each individual, we calculated a core use area (50% isopleth) and home range area (95% isopleth) separately for stationary and commuting segments using continuous-time stochastic process (CTSP) models and autocorrelated kernel density estimation (AKDE) in the R package ctmm^21^. AKDE uses CTSP models to calculate an optimal smoothing bandwidth that accounts for autocorrelation, enabling rigorous comparisons of AKDE home range values between individuals with different movement patterns, sampling intervals, telemetry errors, and sample sizes^27^. We fit models incorporating telemetry location error and selected among competing models using the Akaike Information Criterion corrected for small sample size (AICc^28^;). Because the stationary and commuting segments were spatially and temporally distinct from one another, we calculated home ranges for both segments reasoning that a home range estimate just for the stationary segments would greatly underestimate total area traversed, but including all segments for one overall home range would greatly inflate the area used during non-commuting segments. Therefore, we estimated both home ranges (for commuting birds) and used a paired t-test to compare home range area (log transformed) between stationary and commuting segments.

### Recursion statistics

To characterize movement behavior during commuting segments we used recursion movement metrics (e.g., returns to a previously visited area). First, we defined a bird’s place of residency as the area within the 95% isopleth of each bird’s home range during the stationary segment. We then used the ‘getRecursionsInPolygon’ function in the R package recurse^29^ to calculate the number of *revisits* to a bird’s place of residency during commuting segments, which indicates the number of commuting trips (i.e., a movement to an area outside of a bird’s place of residency and followed by a return back into the defined area). Additionally, during commuting segments we calculated the time spent outside of an individual’s place of residency, *time to return*, denoting the length of each commuting trip. Last, we calculated *residency time*, the time spent within a bird’s place of residency during commuting segments, which represents the time between commuting trips. Recursion statistics were only calculated for birds that exhibited both stationary and commuting segments and only for locations during commuting phases. We did not include a time threshold (i.e., time outside the defined polygon) for any of the recursion metrics.

We used a two-step process to identify the time of year and movement period (stationary or commuting) when peak nesting occurred. First, using the same recursion metrics as above, we quantitatively identified the most probable nesting location of each female during the 2020 and 2021 breeding seasons. To identify locations frequently revisited from day-to-day, we defined a 20-m radius around each diurnal location and calculated the number of *revisits* to that area, separated by at least 12 h. Twenty meters was chosen to define a small area such as a nest location but account for GPS location error (approximately 3 × the average error). We also calculated for each revisit *residency time*, the total time spent inside the 20-m radius, and *return time*, the time between revisits. The most probable nest location was defined as the location with the highest number of revisits, as well as the longest residency time and shortest return time. Second, to identify the timing of peak nesting, we calculated the same recursion metrics for the nest location identified in the previous step for each individual with a 50-m radius around that location and no time threshold. The slightly larger 50 m was to capture use of perches and trees adjacent to the nest that can be used while birds are physically attending a nest but not actually on the nest. Although both males and females incubate their eggs, males rarely incubate during the period around hatching whereas females brood chicks day and night for the first few weeks^10^. Therefore, all movements entering and exiting the 50-m radius circle around the estimated nest location were counted to determine the number of *revisits* and the *residency* and *return time* associated with those visits. We then defined the most likely date around hatching as the period when the residency time peaked above 90 h (approximately 3–4 days) and return times were near zero. We based these thresholds on observations that females can spend as much as 92% of their time on nests brooding in the first week of nestlings hatching^10^.

### Habitat selection

‘Io are found in a variety of habitats^12^that can be broadly classified into four categories: (1) forest, defined as intact, native dominated forest (wet and montane); (2) shrub-grassland, which in many cases is forestland converted to more open habitat; (3) agriculture, areas of fragmented forest and open lands or orchards; and (4) developed, which could include urbanized areas. For all ‘Io locations, we used the Carbon Assessment of Hawaii (CAH) Land Cover and Habitat Status Maps (30 m resolution^30^;) to extract the percent cover of forest and disturbed areas (both agriculture and urban development). We calculated the percent cover of both habitat features at a local and landscape scale by calculating the proportion of pixels within a 40-m (local) and 500-m (landscape) circle around each location. We chose 40 m to be slightly larger than the 30 m pixel habitat classification layer to capture heterogeneity in habitat at the local scale (i.e., edges), and the 500 m neighborhood buffer was chosen a priori to encompass ½ km of habitat coverage around each location, a scale we believed would capture a reasonable extent of landscape that an ‘Io might select from one step to the next. We also extracted the landcover type at each ʻIo location from the CAH Land Cover Map, which we grouped into the four habitat categories (forest, shrub-grassland, agriculture, developed), as well as barren and water. However, all locations that were classified as water were removed from the dataset given that ʻIo are not known to fly over open water^19^, and we excluded locations in barren habitat given few observations in this habitat type.

We examined potential differences in habitat selection of ‘Io within stationary and commuting areas using an Integrated Step Selection Analysis (iSSA)^31^. iSSA accounts for autocorrelation of locations and temporally varying availability distributions resulting from movement constraints by simultaneously making inferences about both habitat selection and the movement process^32^. To evaluate habitat selection, we split individual-level data based on locations in different movement states. We only retained individual datasets that included > 50 locations or steps after splitting the data and maintained a median sampling interval of 2 h. For each individual, we characterized the observed movement patterns using a gamma distribution of their step lengths (i.e., straight-line distance between two consecutive locations) and a von Mises distribution of their turning angles (i.e., angular deviations between headings of two consecutive locations), separately for stationary and commuting areas. We then matched each observed step with 30 available steps randomly sampled from the observed step length and turn angle distributions^33,34^. Each model included four landcover covariates (forest cover and disturbed cover at the local and landscape scale), intersected at the 30-m pixel where each step ended. In addition, for individuals that occurred in all four landcover classifications (forest, shrub-grassland, agriculture, and developed) (Fig. S1), we included landcover type as a covariate in the model with forest as the reference level. We scaled and centered all covariates prior to model fitting. We also included the step length, logarithm of the step length, turning angle, and the cosine of the turning angle in each model to account for the underlying movement process^31^.

Attributes (e.g., habitats, step length) of observed steps were compared with randomly generated ‘available’ steps using conditional logistic regression, with iSSA models fitted to each individual separately for locations in stationary and commuting areas using the R package ‘amt’^35^. To evaluate consistency in ʻIo movement related to habitat features, we summarized individual selection of habitat features and movement responses based on model coefficients and their 95% confidence intervals (CIs). We focused on population-level inferences to avoid potential within-individual autocorrelation that may lead to small estimates of uncertainty. We used inverse-variance weighted regression to summarize across individual model coefficients to obtain population-level averages and 95% CI for each area. Confidence intervals were calculating using empirical bootstrapping which accounted for availability and the uncertainty in each individual’s response^32,36,37^. Parameter estimates for percent cover covariates express the relative selection strength on the log scale (log-RSS^31,33^;) for a 1-unit increase in the specified covariate, where positive values express selection and negative values express avoidance. Log-RSS values for each landcover type were in reference to forest habitat where positive values indicate selection for the specified landcover type over forested habitat while negative values indicate avoidance of the specified landcover type compared to forested habitat. We interpreted CIs overlapping with zero as indifference, and nonoverlapping CIs as significant selection or avoidance of the variable of interest. To understand movement patterns in both areas irrespective of habitat selection, we calculated selection-free step length and turning angle distributions for each individual using the iSSA model parameter estimates of ln(step length) and cosine turning angle, respectively, to adjust the initially observed von Mises and gamma distributions^31^. We also calculated the mean movement rate for each individual in both areas by multiplying the adjusted shape and scale parameters from the gamma distribution.

## Results

Fifteen ‘Io were captured at multiple locations across the eastern side of the Island of Hawai‘i (Fig. 1) and tracked for an average of 508 days (range: 139–563) with an average of 7,421 estimated locations per bird (range: 2,384–9,523; Table 1). Six were captured in urban, developed areas (5 females, 1 male), 5 in rural agriculture areas (4 females, 1 male), and the remaining 4 in forested areas (all females). We detected birds on average for 97% ± 0.03 (standard deviation, SD) of the days a bird was tracked with an average of 15.3 ± 2.1 (SD) locations per day.

Our analysis indicated that many of the ‘Io did not show range residency, but in fact exhibited periods of movement outside their home range. Using segmentation analysis, we detected change points in the movement tracks of 80% of the ‘Io tracked, averaging 4.1 segments per bird (range: 2–7; Fig. 2). The majority (n = 10) of ‘Io had change points associated with large shifts in the variance of locations as birds alternated between two distinct movement states: one state characterized by relatively constrained movements within a place of residency, and a second state characterized by frequent back and forth movement between their place of residency and other noncontiguous locations (Fig. 3A). We termed these movement states as stationary and commuting, respectively. Track segments classified as commuting had significantly larger standard deviation of Easting and Northing locations than segments classified as stationary (Easting locations: F_1,39.5_ = 143.1, p < 0.001, Northing locations: F_1,39.5_ = 163.1, p < 0.001). Similarly, the home range area of individual ‘Io during commuting periods was 19-fold larger than that during stationary periods (t = −6.4, df = 9, p < 0.001) (Table 2; Fig. 4). Five out of 15 ‘Io did not exhibit commuting behavior during the tracking period. Three had a single segment throughout their tracking period that consisted of short distance movements within a confined space that was comparable to the stationary period in the other tracked birds (Fig. 3B). The final two birds also exhibited constrained movement within a confined space, but exhibited a small shift in spatial use within their confined area during the period we tracked them (Figs. S3l [b13] and S30 [b16]).

**Fig. 2:**
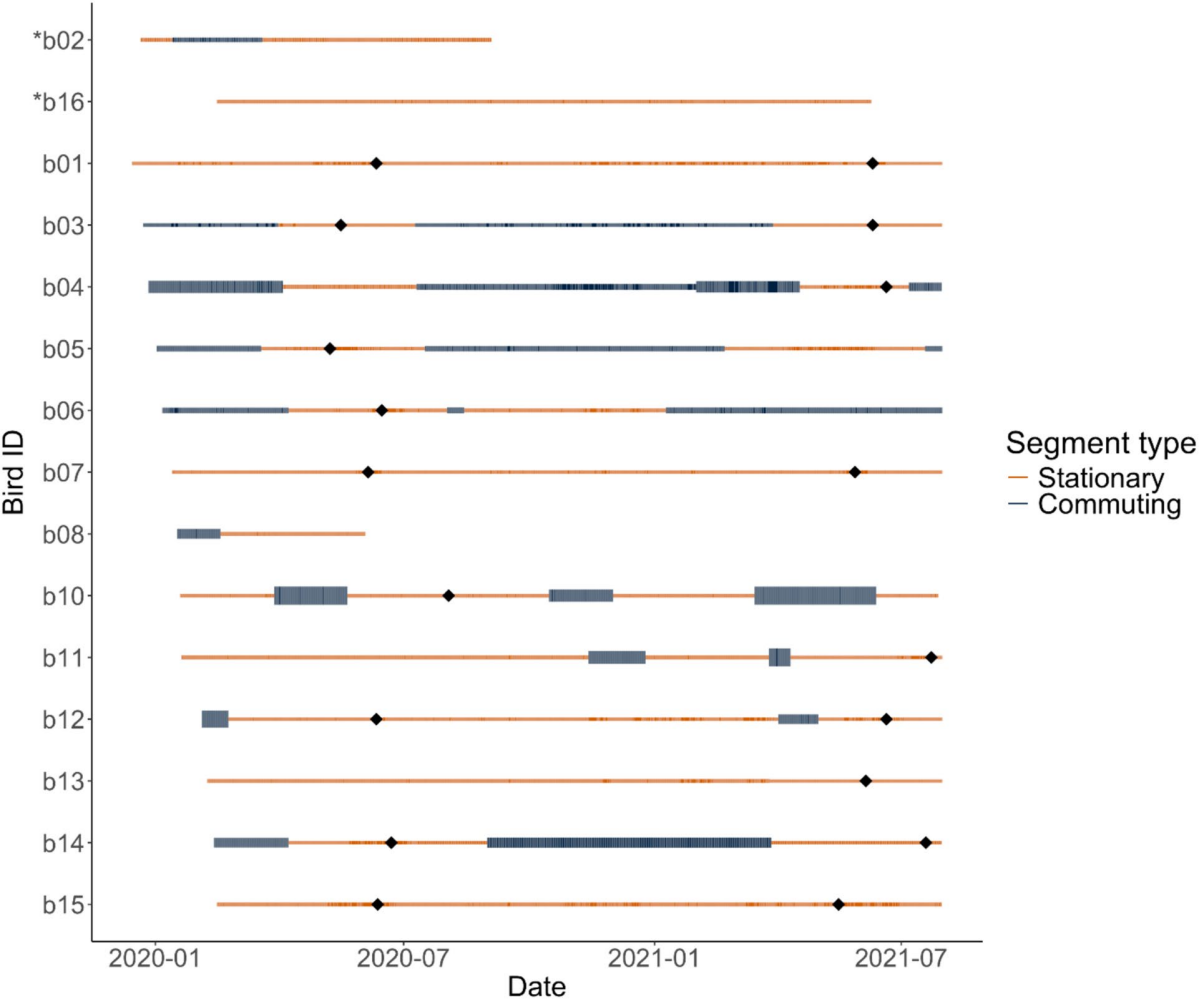
Duration of individual ‘Io (*Buteo solitarius*) movement data collected with GPS-GSM tags on the Island of Hawai‘i, 2019–2021. Tracks were segmented based on shifts in either mean location or variance of locations and then categorized as stationary and commuting time periods. The width of the bar for each segment phase is proportional to the average standard deviation of Easting and Northing locations during that segment. Black diamonds represent the estimated time when hatching occurred for nests of females based on a large increase in the time spent at the presumed nest site location. Asterisks indicate males (b02 and b16).

**Fig. 3:**
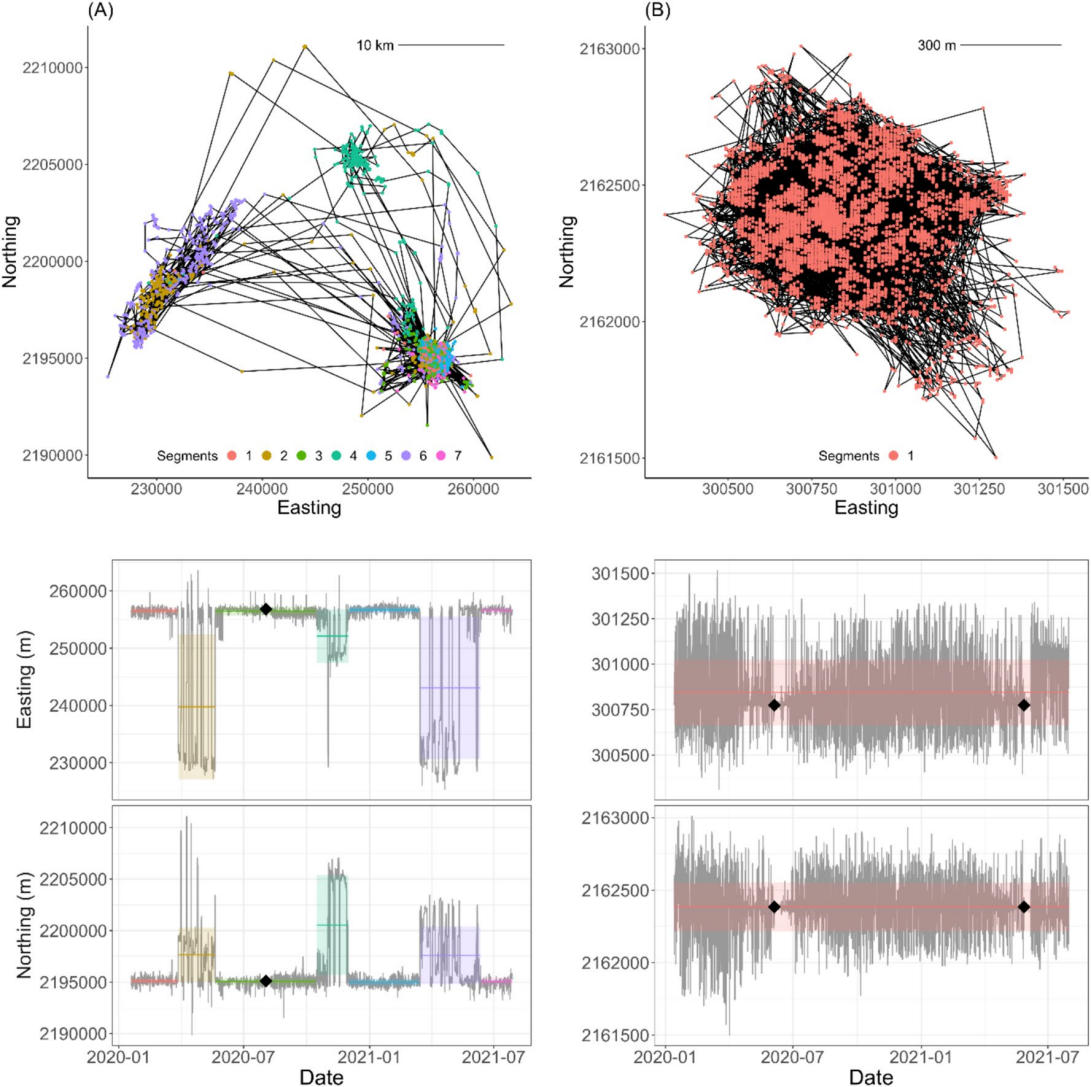
Segmentation of individual ‘Io (*Buteo solitarius*) movement data based on shifts in either mean location or variance of locations for (**A**) an ‘Io (b10) with multiple segments during her tracking period classified as stationary (segments 1,3,5,7) or commuting (segments 2,4,6), and (**B**) an ‘Io (b07) with only a single stationary segment. Top panels show a representation of the bird’s movement path for the duration of tracking with points colored by track segment. The bottom panels represent the corresponding time series of location coordinates (Easting and Northing) for each bird, with a black diamond marking the estimated date of hatching based on a large increase in the time spent at the presumed nest site location. The colored bands over the time series show the estimated mean (horizontal line in the middle of the band) ± standard deviation (band width) of each segment to provide indication of relative variability among segments.

**Table 2:** Home range (95% isopleth) and core area (50% isopleth) estimates, along with 95% confidence intervals, of ‘Io (*Buteo solitarius*) tracked with GPS-GSM transmitters on the Island of Hawai‘i, 2019–2021, calculated separately for track segments classified as stationary or commuting. In addition, recursion statistics characterizing movement behavior of birds during commuting segments are presented, along with minimum and maximum values. Recursion statistics were based on movements outside of bird’s place of residency defined by a bird’s home range area during stationary segments. The variables included are the number of times a bird commuted from their stationary place of residence (No. commutes), the time between commuting trips, how long a bird was away from their place of residency (Duration of commutes), and the proportion of total tracked time movements were classified as commuting.

	Home range area (ha)		Core area (ha)		No. commutes	Time between commutes (hr)	Duration of commutes (hr)	Proportion time commuting
Bird ID	Stationary	Commuting	Stationary	Commuting				
b01	77.2 (75.1–79.3)	–	15.8 (15.3–16.2)	–	–	–	–	–
b02	194.6 (186.9–202.4)	611.2 (559.8–664.9)	32.0 (30.8–33.3)	99.5 (91.2–108.3)	25	43.4 (0.3–284.1)	3.8 (1.0–21.7)	0.09
b03	11.9 (11.4–12.4)	146.1 (138.1–154.3)	0.4 (0.3–0.4)	28.7 (27.1–30.3)	259	11.6 (0.1–2,732.3)	9.6 (1.0–187.7)	0.30
b04	1,507.6 (1,383.0–1,637.4)	25,587.2 (20,812.4–30,847.9)	287.5 (263.8–312.3)	4,209.4 (3,423.8–5,074.8)	43	47.3 (0.3–2,523.3)	26.6 (1.0–925.2)	0.21
b05	288.9 (276.0–302.0)	1,992.8 (1,868.5–2,121.0)	30.3 (28.9–31.7)	235.6 (221.0–250.8)	181	21.3 (0.4–3,592.3)	3.8 (1.0–86.4)	0.14
b06	216.2 (203.9–229.0)	662.9 (625.0–701.8)	35.7 (33.6–37.8)	94.7 (89.3–100.2)	98	41.9 (1.1–426.7)	19.5 (1.1–3,293.7)	0.52
b07	53.6 (52.1–55.2)	–	10.1 (9.9–10.4)	–	–	–	–	–
b08	240.5 (220.4–261.4)	9,373.9 (7,130.2–11,919.7)	45.7 (41.9–49.7)	1,511.1 (1,149.4–1,921.5)	13	22.2 (4.0–198.0)	14.5 (1.1–71.9)	0.33
b10	392.8 (374.2, 411.9)	53,862.5 (42,838.8–66,129.3)	64.5 (61.4–67.6)	11,503.6 (9,149.2–14,123.5)	58	19.5 (0.4–2,520.4)	4.7 (1.1–3,457.6)	0.58
b11	237.9 (229.9, 246.1)	65,926.6 (37,041.0–103,001.7)	59.7 (57.7–61.7)	13,855.0 (7,784.5–21,646.7)	9	45.0 (5.2–2,181.6)	76.9 (1.9–169.5)	0.19
b12	117.2 (113.7, 120.6)	5,338.1 (448.3–6,307.8)	16.8 (16.3–17.3)	957.0 (797.5–1,130.9)	20	19.1 (0.1–9,692.6)	23.9 (1.9–48.9)	0.04
b13	117.2 (112.7, 121.8)	–	19.5 (18.7–20.2)	–	–	–	–	–
b14	231.2 (210.6, 252.8)	3,535.7 (3,211.3–3,875.4)	49.4 (45.0–54.0)	451.5 (410.1–494.9)	83	25.4 (0.2–314.3)	23.7 (1.2–3,211.1)	0.56
b15	309.1 (297.4, 320.9)	–	71.2 (68.5–73.9)	–	–	–	–	–
b16	305.9 (296.0, 316.1)	–	86.84 (84.0–89.7)	–	–	–	–	–
Average	286.8	16,703.7	55.0	3,294.6	79	29.7	20.7	0.30

**Fig. 4:**
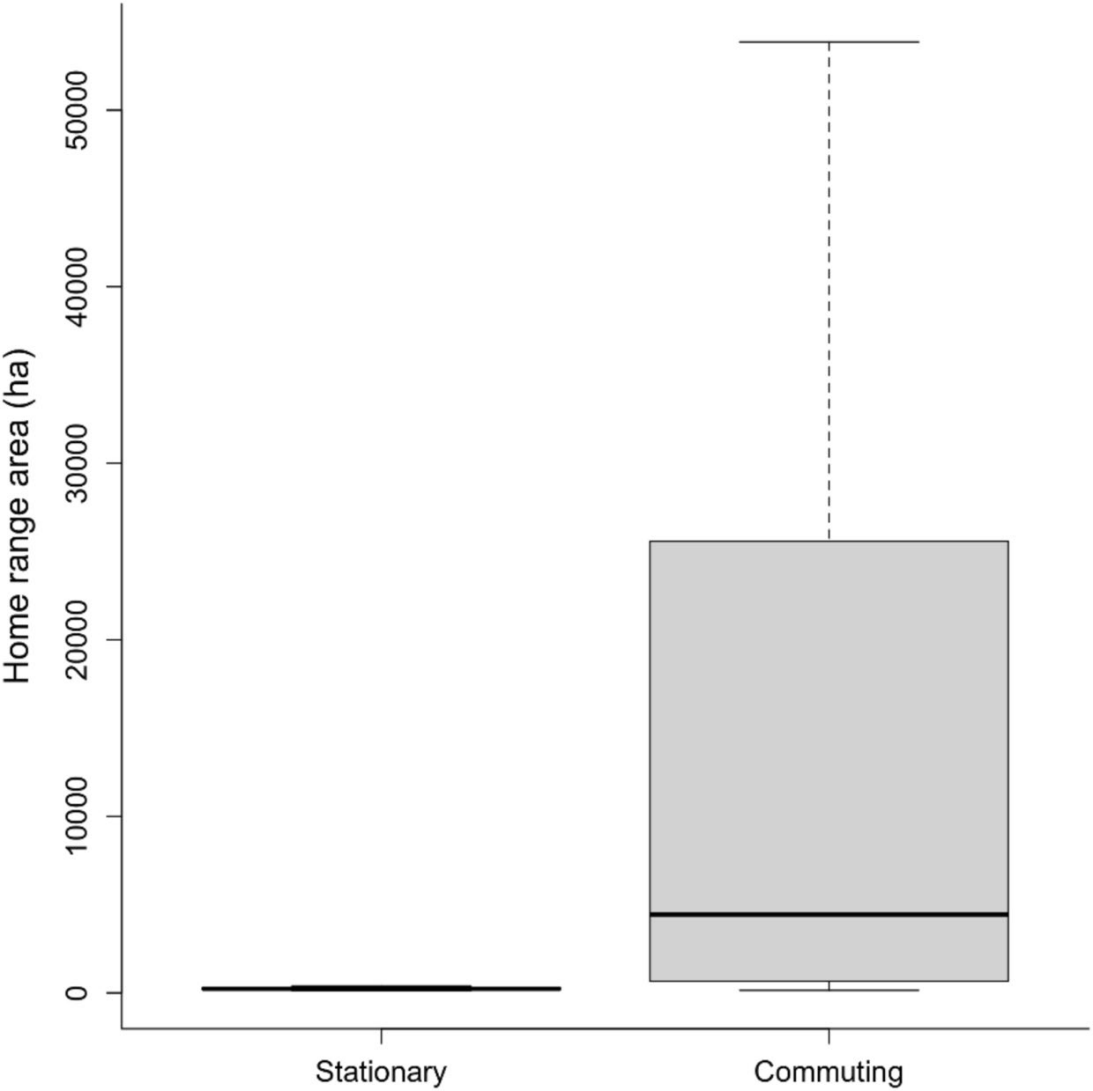
Home range sizes of ‘Io (*Buteo solitarius*) comparing tracking periods classified as stationary or commuting. Box plot whiskers depict the 10^th^ and 90^th^ percentiles and boxes show the 25^th^ and 75^th^ percentiles with median values indicated.

The commuting state represented a distinct shift in movement behavior. Commuting segments lasted a median of 71 days (24–180 days), compared to stationary segments that were the dominant movement behavior state (median 122 days, range 86–269) for the majority of birds. During commuting segments, birds made a median of 50 (range: 9–259) short-duration movements to areas outside of their place of residency, before returning, with the median length of commuting trips lasting 17.0 (range: 3.8–76.9) hours and the time between commuting trips ranging from 11.6–47.3 h (median time: 28.3 h) (Table 2). The average step length between recorded locations was 1,149 m (range: 1–39,105) during commuting trips, compared to only 434 m (1–3,945) during stationary segments (Fig. S1A). Differences in the step length distribution between segment phases resulted in faster average movement rates during commuting segments (1,151 m/2 h, range: 386–1,726) than during stationary segments (520 m/2 h, range: 190–746). Likewise, ‘Io made more directed movements during commuting trips than when residing within their place of residency, where movements were more tortuous (Fig. S1B). In most cases, the commuting destination was a relatively small, finite area, with few locations recorded between a bird’s place of residency and their commuting destination, indicating that commuters in most cases were flying directly to their commuting destination and not exploring areas in between (Fig. 3A).

**Fig. 5:**
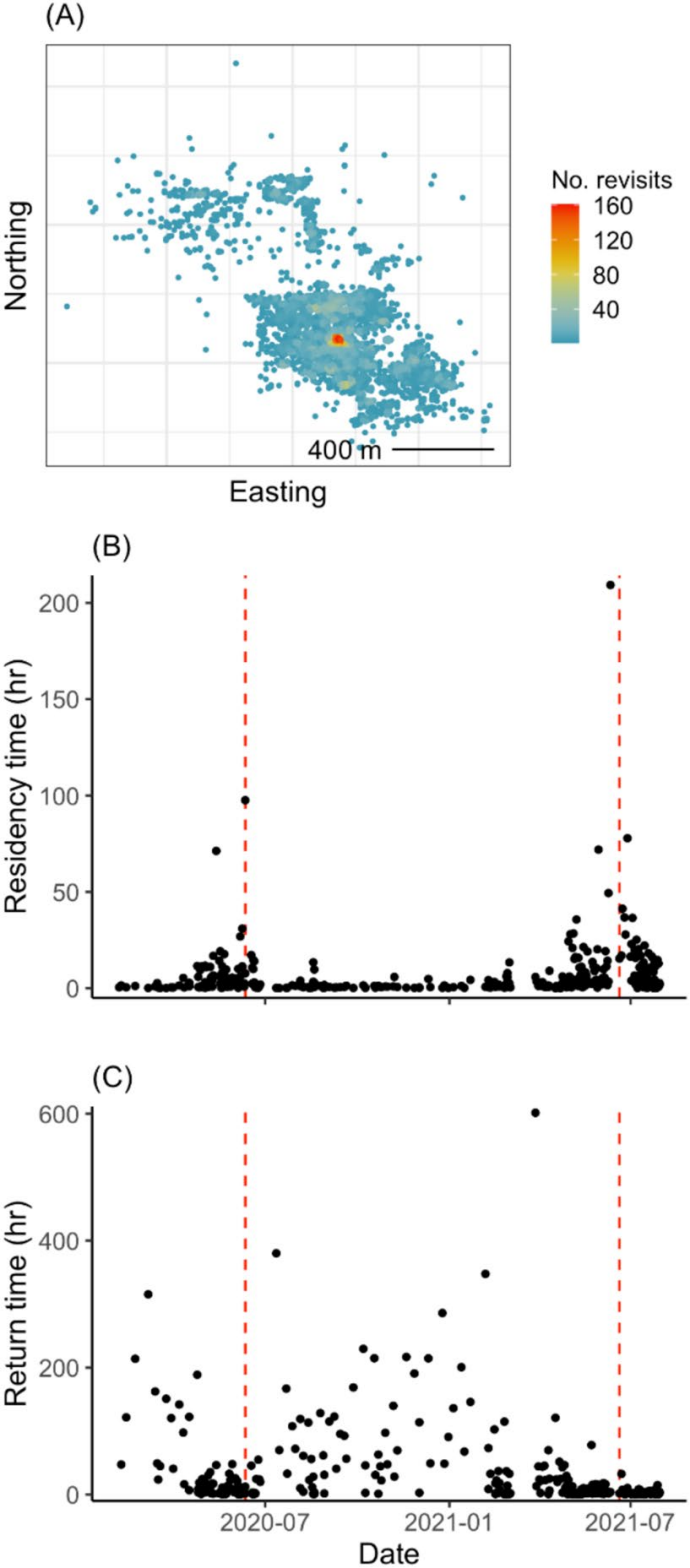
Example of the use of recursion statistics to estimate the time of hatching or peak breeding for female ‘Io (b12; *Buteo solitarius*) tracked on the Island of Hawai‘i, 2019–2021. Panel (**A**) shows the number of revisits to all detected locations (within 20 m and separated by 12 h), with the most probable nest location indicated by the greatest revisits (hot colors). Based on a 50-m diameter around the probable nest location we calculated the residency time (how long they remained within 50 m of probable nest location) of each revisit panel (**B**) and the time to return to the probable nest location when they left panel (**C**). The date with the highest residency time and lowest return time are the estimated hatching or peak breeding time (red dashed lines), consistent with the published field observations that female ‘Io rarely leave their nest when eggs hatch.

We found evidence for nestling care at least once for all females and twice for 50% of the females (Table 1, Fig. 5). All estimated peak nesting activity for female ‘Io occurred during a stationary segment (Fig. 2). The time that females spent at the presumed nest location during the estimated hatching period ranged from 91 to 405 h (~ 4–17 days). For three females the estimated nest location differed between the 2020 and 2021 breeding seasons (Table 1), but the average difference between the nest site locations was only 315 ± 69 m.

**Fig. 6:**
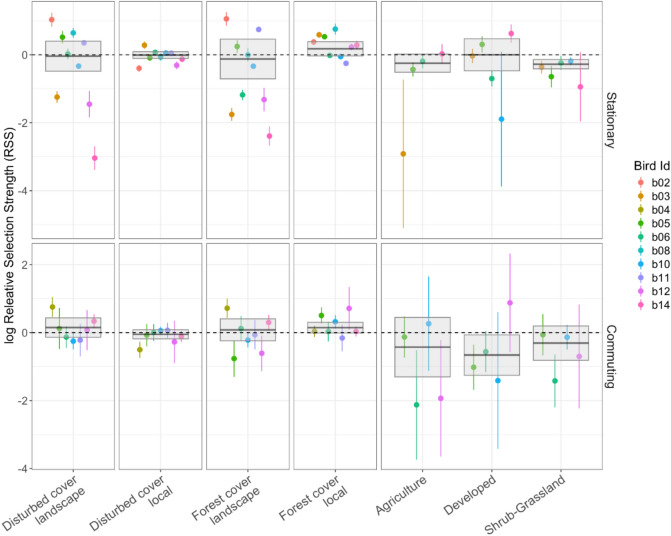
Habitat selection in ‘Io (*Buteo solitarius*) tracked on the Island of Hawai‘i, 2019–2021, during stationary and commuting phases. The points depict individual-level estimates with 95% confidence intervals (CIs) of log-relative selection strength (log-RSS) of different habitat features. Local represents habitat covers within 40 m of a recorded location, and landscape is habitat covers withing 500 m of recorded location. Population-level estimates and 95% CIs of log-RSS are depicted by a solid horizontal line and light gray box, respectively, and were calculated based on inverse-variance-weighted regression on individual models. Log-RSS indicates the strength of selection (if positive) or avoidance (if negative) for either a 1-unit increase in each continuous covariate values (4 left panels) or in reference to forested habitats (right panel). Disturbed cover is agriculture and developed covers combined. We interpreted CIs overlapping with zero (dashed line) as indifference, while non-overlapping CIs indicate significant selection or avoidance of the variable of interest.

Although we hypothesized that birds from distinct habitats (urban, rural mixed agriculture, forest) would largely remain in those habitats, subsequent tracking indicated birds selected a diversity of habitats (Fig. S2) and that the capture location of a bird did not necessarily represent the dominant habitat type they occurred in. We found large variation in individual habitat preferences, in terms of selection or avoidance of different habitat types (i.e., disturbed and forested areas), which may reflect the heterogenous nature of the landscape. High variability across individuals resulted in most population-level 95% CIs overlapping with zero. However, there was also a strong inclination for selection of forest habitat over alternative habitats, indicating a selection for such habitats at a local level. At a landscape level, there was greater variability in habitat selection among individuals compared to the local level, reflecting the heterogeneous nature of habitats at the landscape level. For example, ‘Io at a landscape-scale demonstrated high individual variability in the selection of areas with high forest cover, while individual ʻIo at the local level selected areas of higher forest cover in proportions greater than available within both stationary and commuting areas (Fig. 6). Moreover, ʻIo within stationary areas selected forest habitats over agriculture and shrub-grassland cover types at the population-level, but not developed areas, while ʻIo within their commuting area selected for forest habitats only over developed areas at the population-level (Fig. 6).

## Discussion

Our study revealed two alternative states of movement behavior in ‘Io: a state characterized by small scale movements within a relatively constrained area that represents an individual’s place of residency (stationary state), and for most tracked ‘Io a secondary state where individuals repeatedly moved back and forth between their place of residency to another, geographically disjunct location (commuting state). This partially supports our expectation of small home ranges in ‘Io, as one-third of the tracked ‘Io showed high range restriction and the remaining two-thirds were within relatively constricted place of residency (between commutes) for most of the time tracked. However, the commuting behavior indicates a dynamic aspect to ‘Io movement ecology that was not expected. Commuting behavior was distinct, occurring during periods outside of core nesting activity, with commuting sessions (lasting 24 to 180 days) characterized by frequent back and forth movements to locations outside of a bird’s place of residency, lasting 4–77 h with 12–47 h between commuting trips. Other raptors have larger home ranges and greater movements during the non-breeding period, but different patterns of movement. For example, some Red Kites (*Milvus milvus*) show post-reproduction movement as great as 589 km, but the movement is wandering and continuous for the entire non-breeding period^38^. Golden Eagles (*Aquila chrysaetos*) will also conduct post-breeding movements, but the movements are for sustained periods away from their core breeding area^39^. The commuting behavior we observed in ‘Io represents a cryptic behavior, as the species is considered to be year-round residents with high territory fidelity over multiple years^10^. The lack of detection of commuting behavior in past studies is understandable, given the difficulty of detecting long-distance movements solely from visual resights or short-distance radio telemetry tracking, and highlights the power of continuous large-scale tracking provided by GPS-GSM tracking devices. Studies have shown that lower quality habitat can lead to larger home ranges^18^, and perhaps the satellite sites provide resources that are not available year-round from a bird’s place of residence. Alternatively, areas that have the best attributes for nesting may not be the best locations for hunting, and commuting provides a method to balance both needs, such as seen in European Starlings (*Sturnus vulgaris*)^40^. This commuting behavior in ‘Io may be more common in tropical raptors than is currently known, and future studies of other tropical raptors may illuminate a diversity of movement strategies by this important taxonomic group.

A key question is why ‘Io make distinct shifts from being sedentary within a finite area to engaging in commuting behavior to noncontiguous satellite locations. Home ranges are generally assumed to be areas that maximize fitness through sufficient acquisition of resources^41–43^, and most animals have home ranges that are spatially restricted and geographically contiguous^44–46^. As a year-round resident in an aseasonal tropical environment, ‘Io do not need to move in response to strong seasonal changes in climate. Nonetheless, small temporal patterns in the predictability of resources can influence an animal’s movement^47,48^, and the change in ‘Io movement patterns may be due to changes in food resources, either exploiting temporally abundant resources in another area or depleting resources in their place of residency. However, the repeatability of this behavior and the fidelity to specific satellite areas indicate more than an immediate response to food shortages. Studies have shown that birds will shift foraging locations once they are free from the constraints of breeding, specifically the spatial constraints of maintaining territories and provisioning nests^49,50^. For example, Canarian houbara bustards (*Chlamydotis undulata fuertaventurae*) in the Canary Islands, found that both males and females will increase the size of their home ranges during the non-breeding period, and shift to more human-altered habitat^51^. We found that the movement of females was most constrained during the estimated peak breeding period (when eggs hatch), and there was no commuting behavior during these periods. Thus, the nonbreeding period may represent an opportunity to maximize food acquisition by hunting in areas outside a bird’s place of residency when females are not constrained by the responsibilities of caring for eggs and young at the fixed location of a nest^52,53^. Because our study was biased toward females, with only two males tracked, we do not know if overall males have different patterns than females, which is the case in some raptors especially with large sexual size dimorphism^54,55^.

Most commuting ‘Io demonstrated spatial fidelity to areas outside their place of residency (i.e., a tendency to return to the same general locations), both within a commuting period and across commuting periods when multiple periods were documented. Fidelity to satellite foraging areas has been observed in both terrestrial and aquatic environments where the advantages of familiarity may be far more important than random searches for peak resources in a given year^56,57^. In a cognitive map framework, individuals create a spatial map of experiences with information on resources and threats and form home ranges, places of residency, or specific foraging locations as they gain information about locations that are beneficial to them^48^. Moreover, cognitive maps are likely regularly updated with new experiences, reinforcing bonds to particular locations^58^. For ‘Io, the satellite commuting areas are generally geographically distinct locations, separate from their place of residence, with few locations detected in between, indicating purposeful movement to specific locations and little indication of exploratory behavior. How individuals establish specific satellite locations is unknown. However, commuting locations are presumably areas that birds discovered during exploratory periods (either as juveniles or during periods of low food resources) and are then reinforced through subsequent visits if productive. A tantalizing clue into how ‘Io may establish and reinforce a hunting location is in their interactions with the ‘Alalā (Hawaiian crow, *Corvus hawaiiensis*). Two efforts to reintroduce captive-bred ‘Alalā into the wild, first in the 1990s and more recently during 2016–2020, both resulted in failure in part from multiple ‘Io predation events on ‘Alalā^59,60^. In both reintroduction events, ‘Alalā were released at communal locations and routine feeding kept them anchored to those locations^60,61^. With the exception of a small release in 2016, ‘Io predation on released ‘Alalā did not occur until almost 2-years post-release, but then continued with regularity^59,60^. The sequence of events suggests that the discovery of concentrated prey, in this case reintroduced ‘Alalā, may be delayed through the high fidelity of ‘Io to hunting locations, but ‘Io may quickly shift focal hunting grounds once an area of concentrated food is identified.

We designed our study to sample ‘Io from three distinct habitats (forested, developed [rural, agricultural], and urban environments) because ‘Io are frequently detected in all of these habitats^11^. We expected differences in movement behavior across the habitat types, particularly if birds were choosing patches of higher habitat quality. However, we found that the home range of many ‘Io encompassed multiple habitat types, and the location where an ‘Io was captured did not necessarily represent the dominant habitat type they selected during the course of the tracking period. Rather, our study indicated high variation in habitat selection among ‘Io, with ‘Io appearing able to persist in highly fragmented habitats. We were not monitoring reproductive success or stress levels, and it is possible ‘Io in fragmented habitat may have had reproductive costs and increased physiological stress, as seen in Northern Saw-whet Owls that occupied areas with high habitat fragmentation^17^. Our habitat selection analysis indicated that at a landscape level, ‘Io did not select large forest tracts (i.e., high forest cover within 500 m of a detected location). However, at a local level, ‘Io do select for forest cover regardless of the larger landscape configuration in which the bird ranged. For example, in highly fragmented urban landscapes, ‘Io selected small forest patches embedded within the urban landscape. The localized selection may indicate that individuals are able to compensate for reduction of forest habitat, but increased movement to reach widely spread forest patches could increase energy costs and reduce fitness^62^. We also found that habitat selection patterns were not necessarily similar between areas of residency and commuting satellite areas, indicating that habitat choice may be more defined by resource concentration in a given area than specific habitat characteristics. Many Buteos are open-country hunters, and ‘Io will readily move across large unforested areas. However, ‘Io primarily use a sit and wait approach to hunting, where they perch above the ground waiting to sight potential prey^10^. Thus, forest habitat may provide the vertical structure necessary to maximize success using this hunting approach to capture passerine birds or introduced small mammals^63^, making forests optimal for foraging success.

The occupancy of multiple, noncontiguous areas by most ‘Io presents both challenges and opportunities for their conservation. A unified conservation strategy is much more difficult for birds that span large geographic areas across different habitats and are managed by multiple entities (federal, state, and private landowners). Ultimately, the viability of a population is driven by the cumulation of all the resources and threats from all areas they occur in^64^. Overall, we found a high level of individual variation in ‘Io movement patterns and habitat selection, which makes generalizations across the species difficult^65^. High individual variation also means that individuals will likely be differentially affected by changes across the landscape (e.g., habitat loss, forest die off). Although the occurrence in multiple disjunct locations may indicate the ability to adjust foraging areas when needed, the consistency of movements between specific commuting sites and a bird’s place of residency indicates the importance of specific locations, particularly forested areas within the landscape. Familiarity with specific locations may be paramount for success in ‘Io, which would make the loss of commuting or residency locations difficult for individuals to recover from. On the other hand, the occurrence in multiple noncontiguous areas indicates a level of spatial flexibility that may allow ‘Io to adapt to the loss of habitat in particular areas, especially satellite areas, or the discovery of new food resources, such as the case of the ‘Alalā. The long-term fidelity to satellite areas is unknown; however, the GPS-GSM tags we placed on ‘Io for this study have continued to function beyond the period analyzed in this study, and for some individuals we will eventually have multiple years of tracking data to evaluate site stability over time.

## Supplementary Information

Supplementary Information.

## Data Availability

All data used for this study are available at: Paxton, E. H., and K. L. Paxton. 2024. ‘Io Hawaiian hawk GPS tracking locations, Island of Hawai‘i, 2019–2020. US Geological Survey data release, 10.5066/P142XU4S.
