# Supplementary material for: Alternating movement strategies of a tropical raptor
Source: Sci Rep. 2025 Aug 13;15:29719. doi: 10.1038/s41598-025-11248-8 (PMC12350784; doi:10.1038/s41598-025-11248-8)
Supplement: Supplementary file 1 — Supplementary Information. [file 41598_2025_11248_MOESM1_ESM.docx]

**Alternating movement strategies of a tropical raptor**

Eben H. Paxton and Kristina L. Paxton


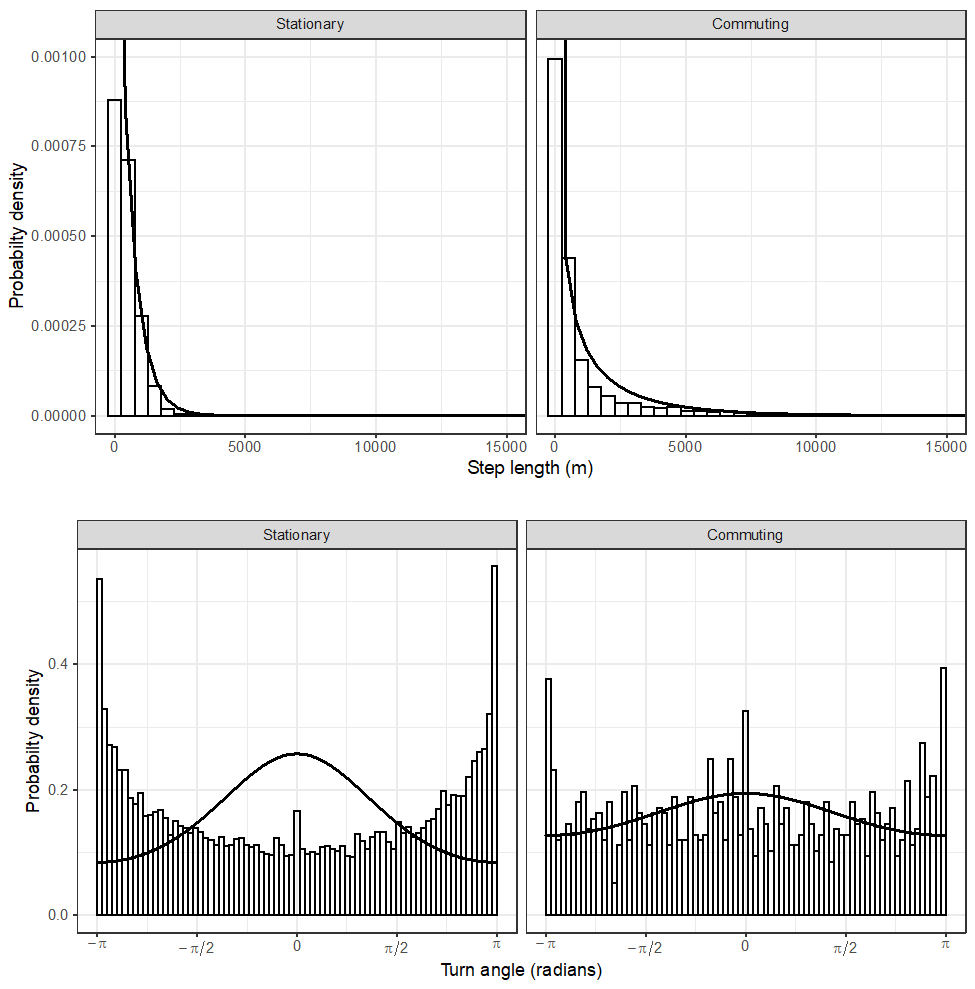


Figure S1. Step length (top panel) and turning angle (bottom panel) distribution for movements of ‘Io (*Buteo solitarius*) during stationary and commuting phases. The black curves depict selection-free step length and turning angle distributions based on integrated step selection analysis (iSSA) model parameter estimates used to adjust the initially observed von Mises and gamma distributions. Selection-free distributions are overlaid on histograms of the raw data. To better visualize the distribution of step lengths, the x-axis was truncated at 15,000 m given the small number of steps lengths during commuting phases that extended beyond this value (maximum step length was 39,105 m).


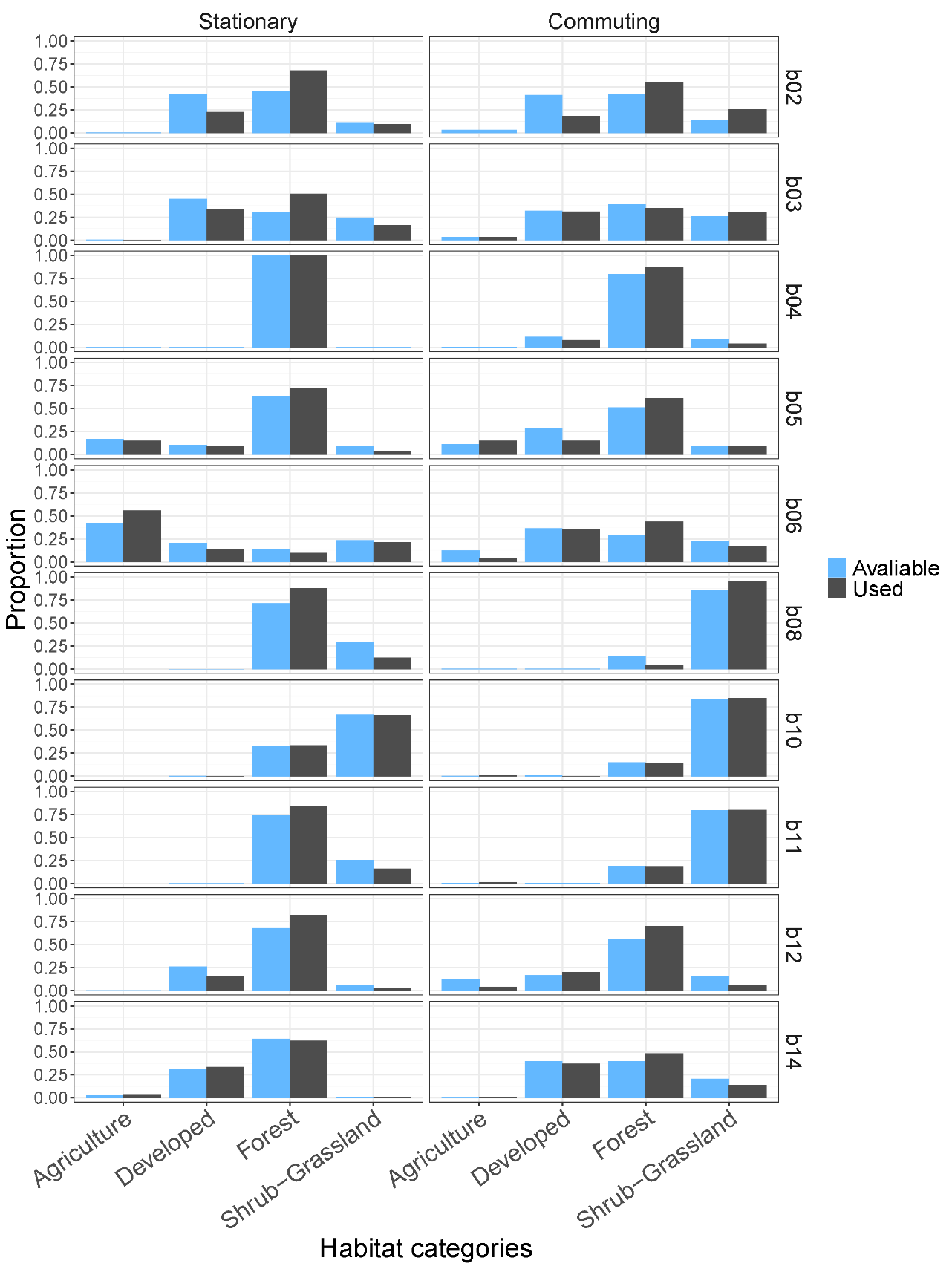


Figure S2. Proportion of observed (used) and random (available) locations or steps of tracked ‘Io (*Buteo solitarius*) associated with each landcover type for locations within an individual’s stationary and commuting use area.

**Figure S3.** Segmentation of individual ‘Io (*Buteo solitarius*) movement data from the Island of Hawai‘i, 2019–2021, based on shifts in either mean location or variance of locations. For each ‘Io, top panels show a representation of the bird’s movement path for the duration of tracking with points colored by track segment. The bottom panels represent the corresponding time series of location coordinates (Easting and Northing) for each bird. The colored bands over the time series shows the estimate mean (horizontal line in the middle of the band) ± standard deviation (band width) of each segment.


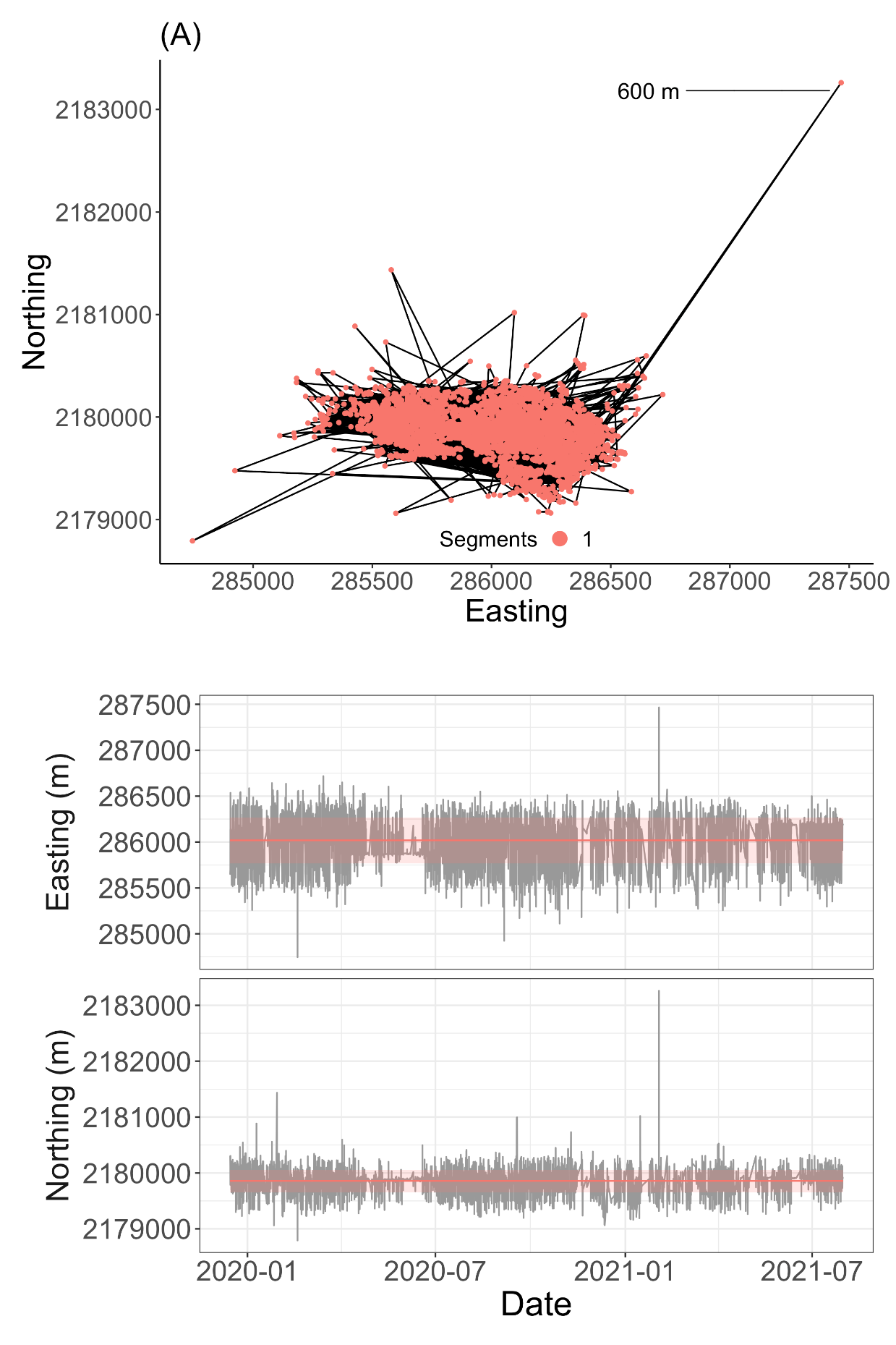


Figure S3a. Movement path (top panel) and corresponding time series of location coordinates (Easting and Northing, lower panels) for ‘Io B01.


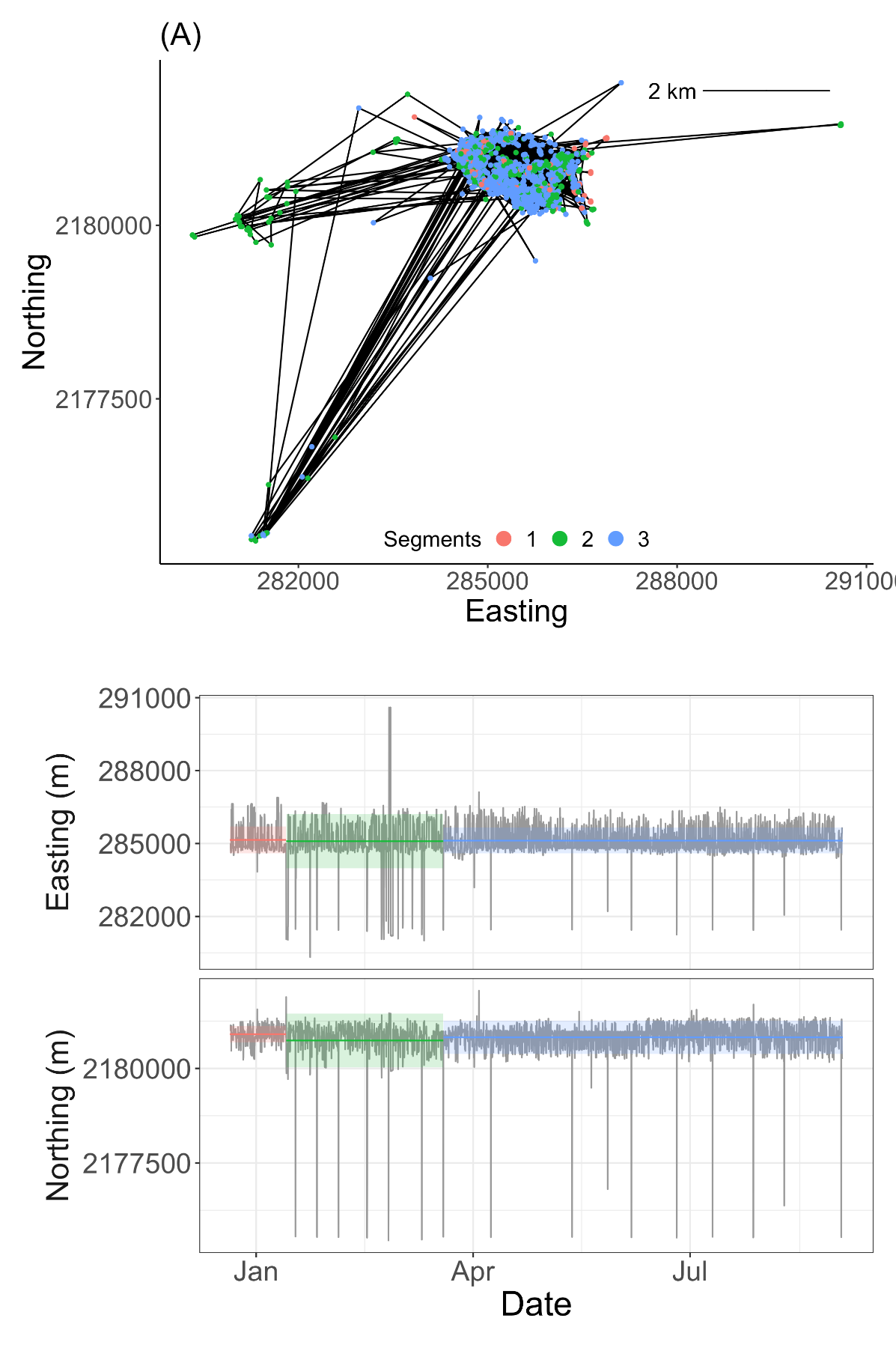


Figure S3b. Movement path (top panel) and corresponding time series of location coordinates (Easting and Northing, lower panels) for ‘Io B02.


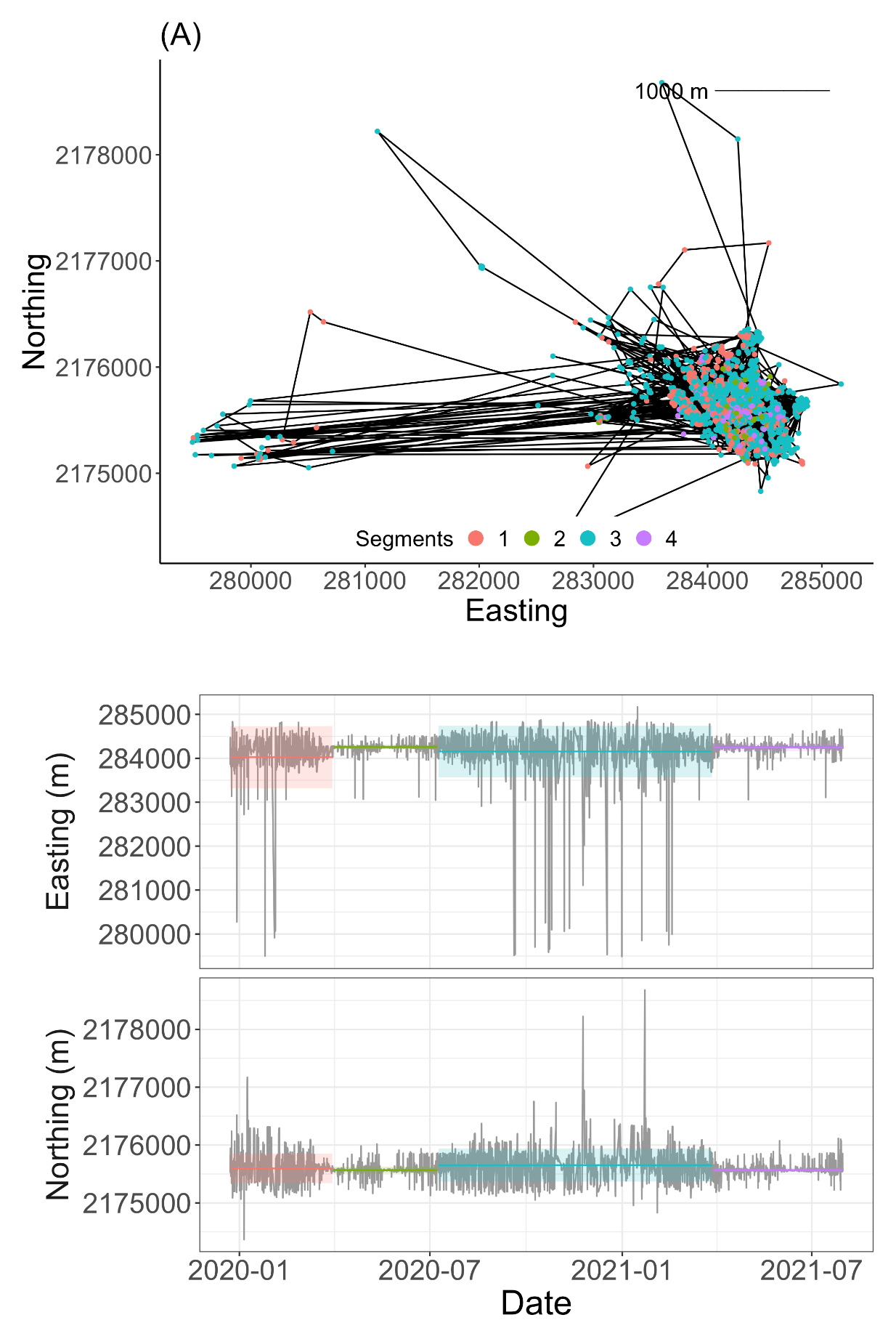


Figure S3c. Movement path (top panel) and corresponding time series of location coordinates (Easting and Northing, lower panels) for ‘Io B03.


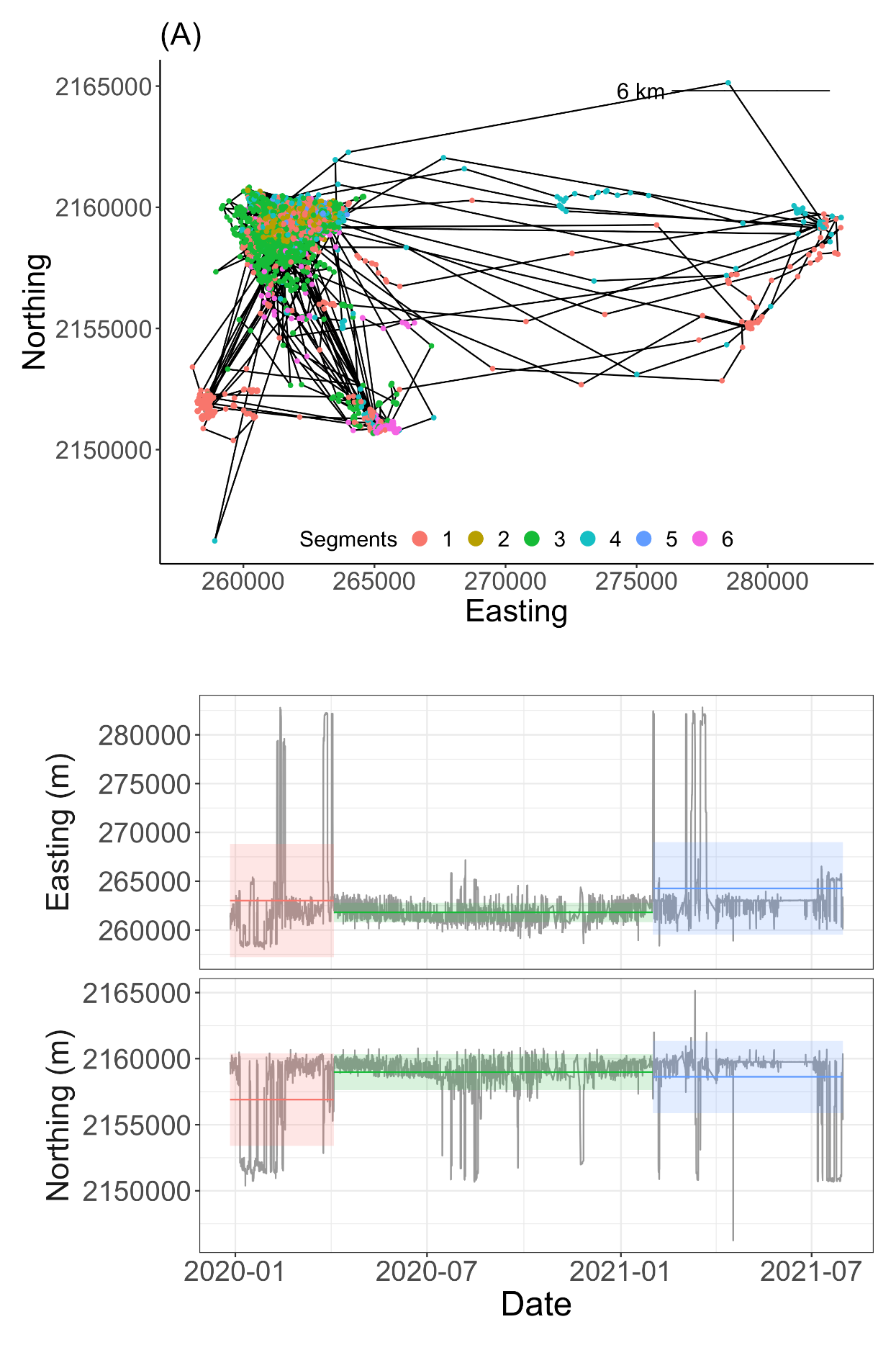


Figure S3d. Movement path (top panel) and corresponding time series of location coordinates (Easting and Northing, lower panels) for ‘Io B04.


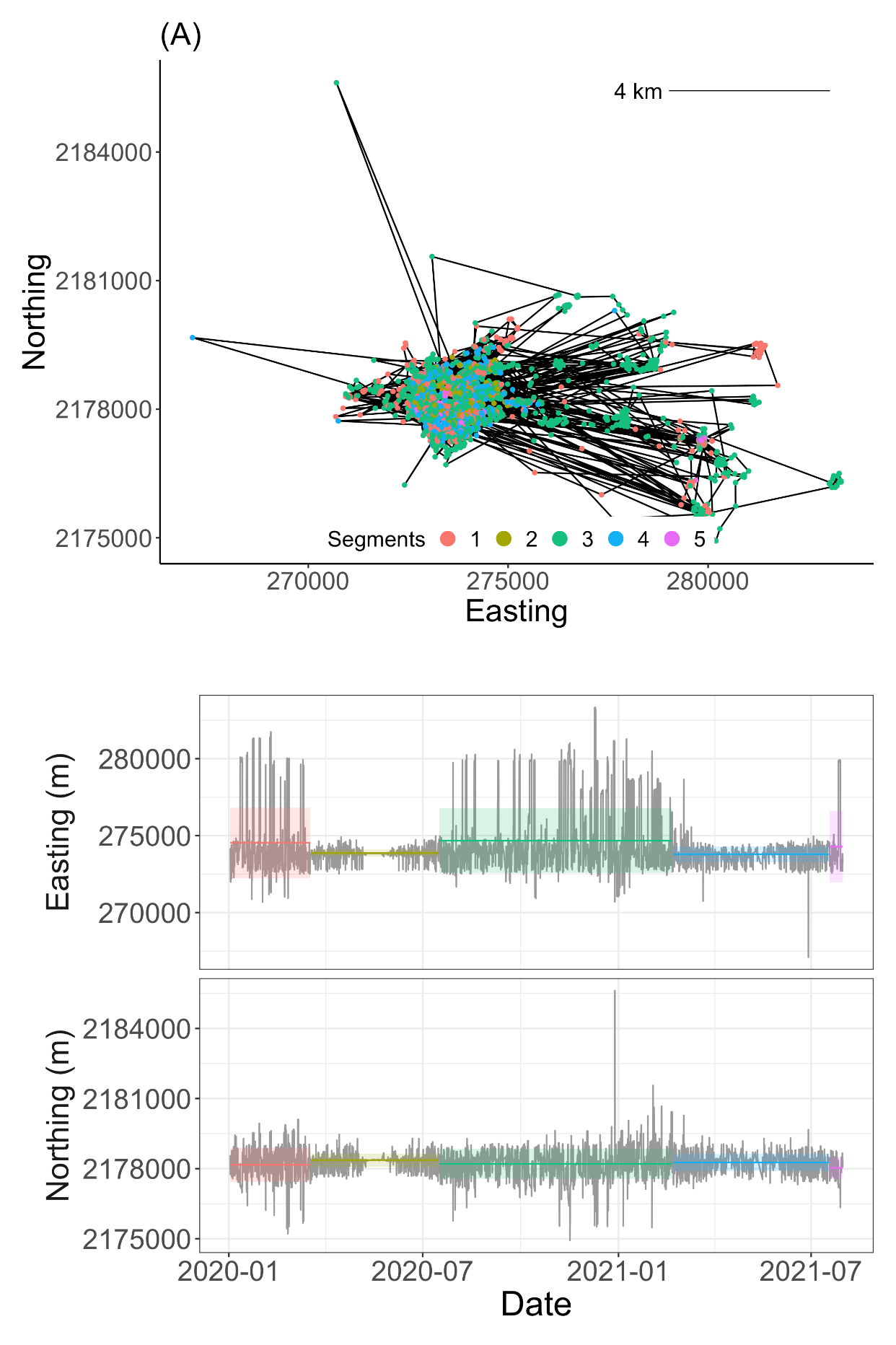


Figure S3e. Movement path (top panel) and corresponding time series of location coordinates (Easting and Northing, lower panels) for ‘Io B05.


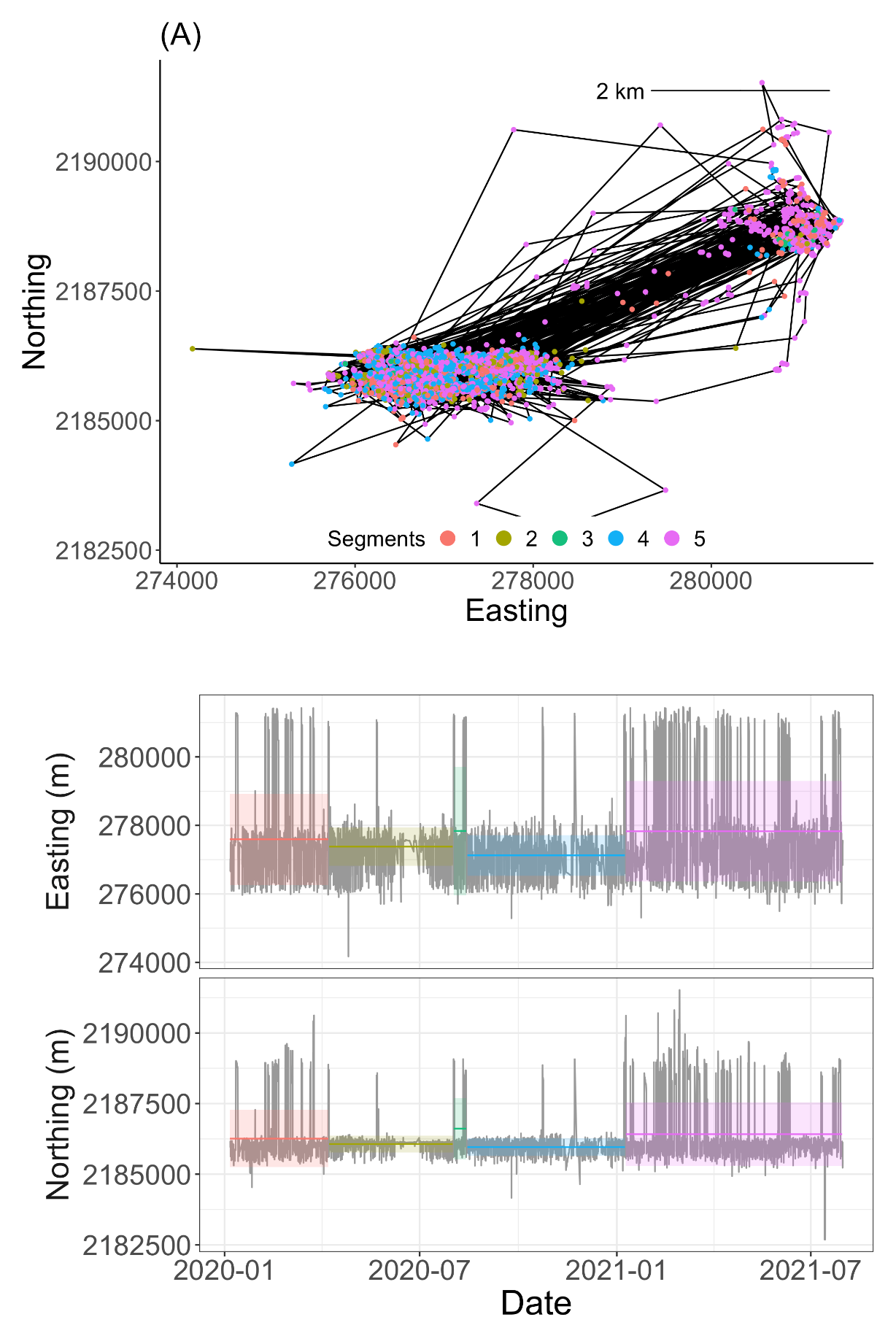


Figure S3f. Movement path (top panel) and corresponding time series of location coordinates (Easting and Northing, lower panels) for ‘Io B06.


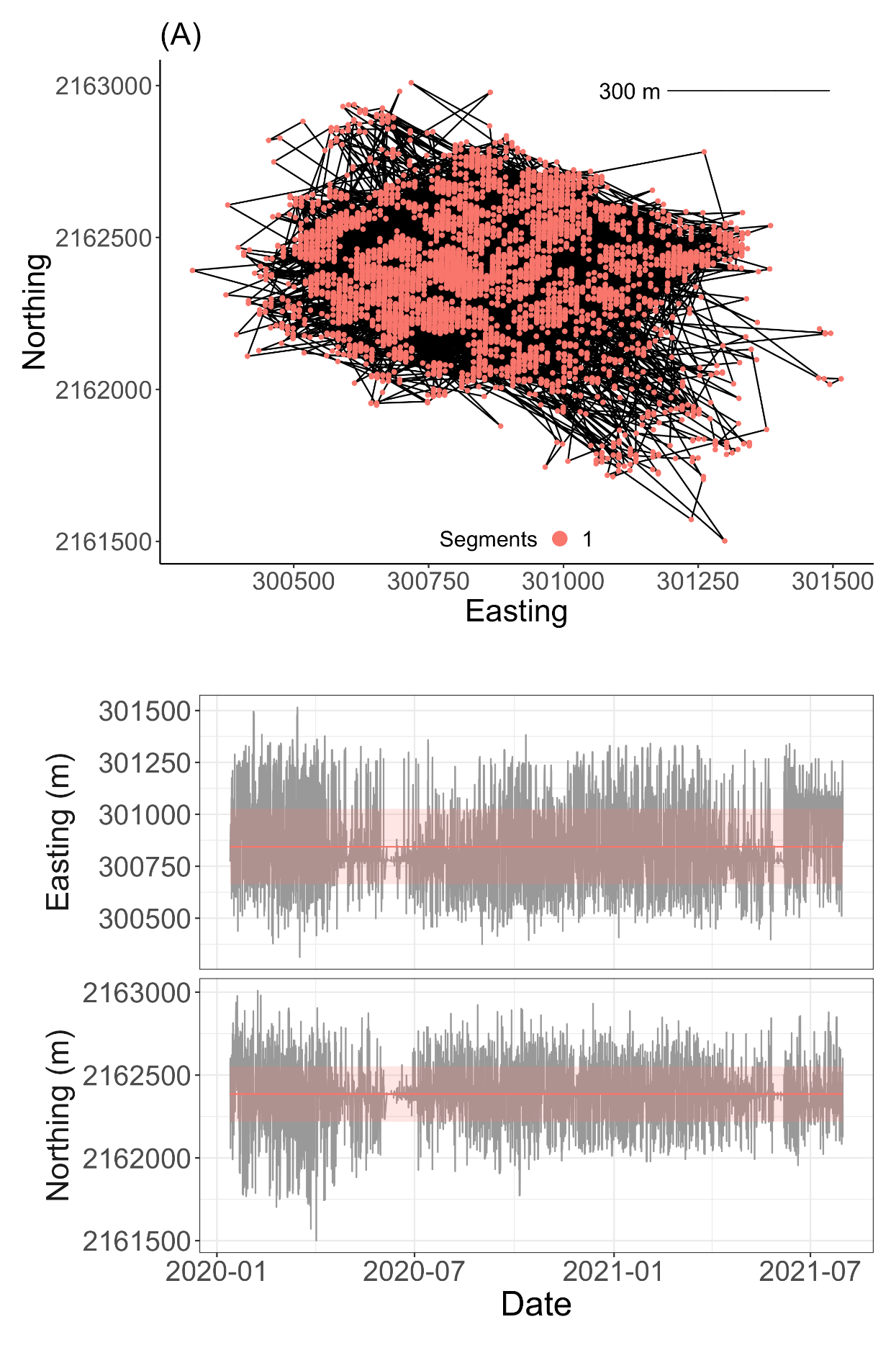


Figure S3g. Movement path (top panel) and corresponding time series of location coordinates (Easting and Northing, lower panels) for ‘Io B07.


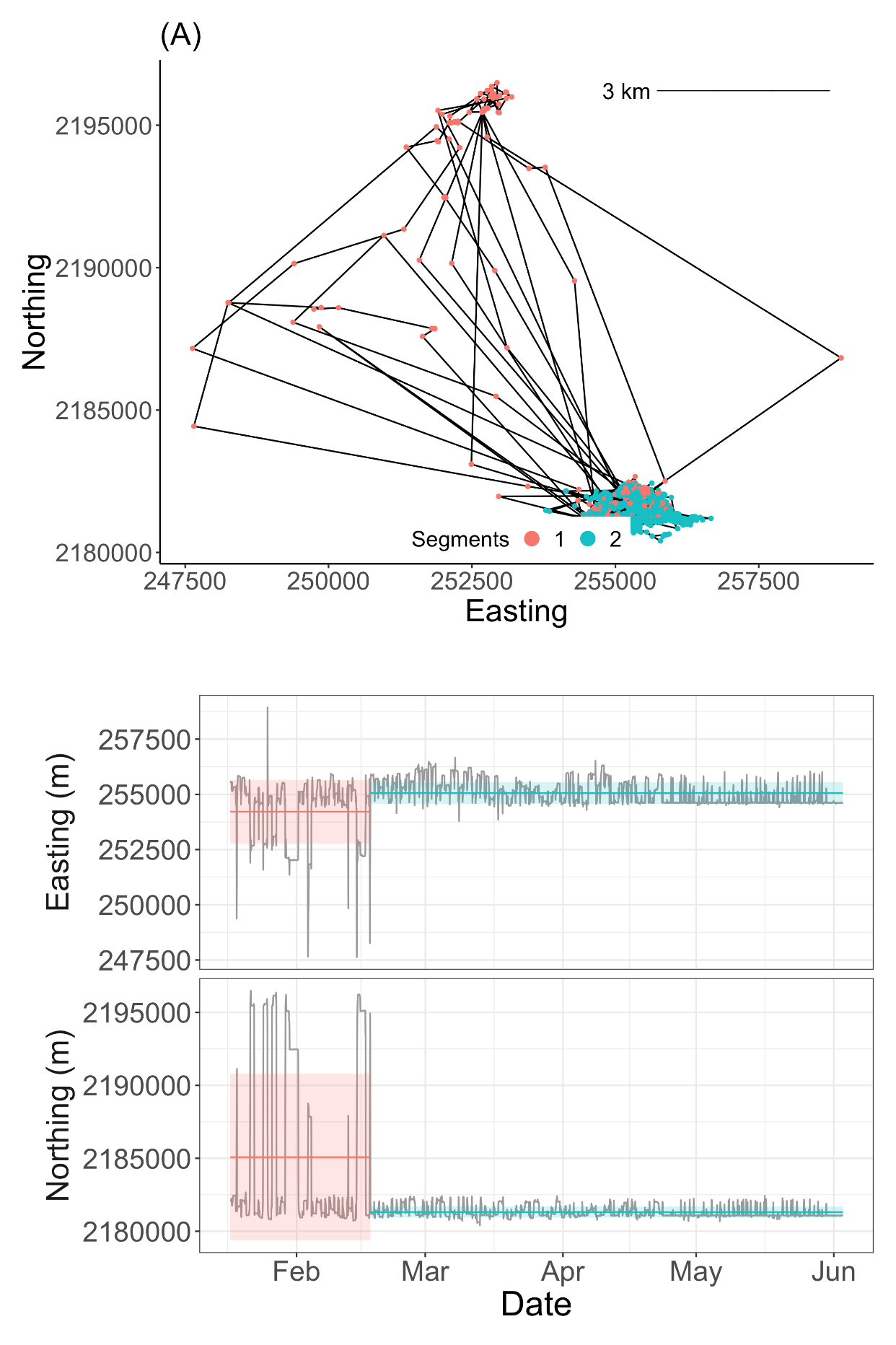


Figure S3h. Movement path (top panel) and corresponding time series of location coordinates (Easting and Northing, lower panels) for ‘Io B08.


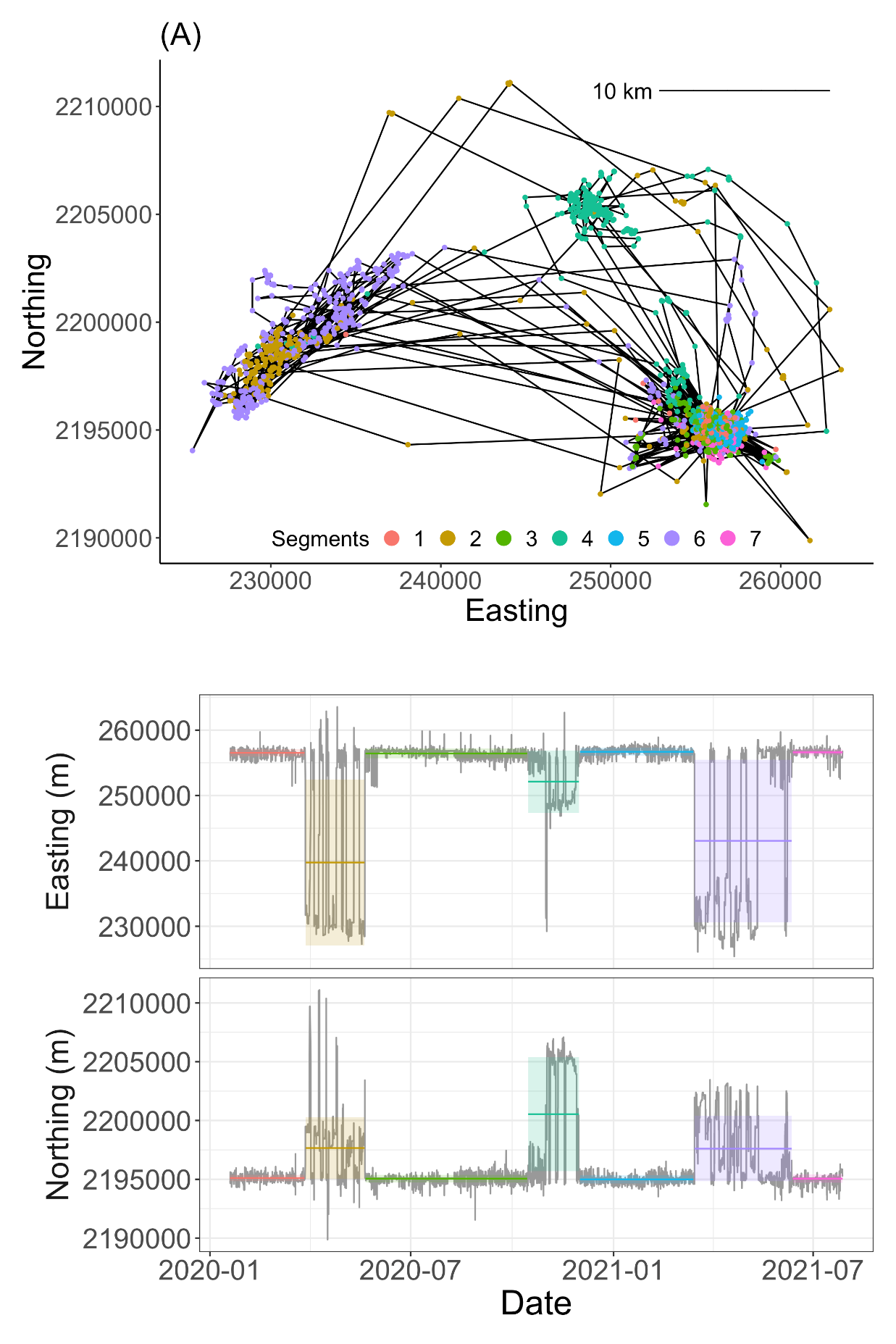


Figure S3i. Movement path (top panel) and corresponding time series of location coordinates (Easting and Northing, lower panels) for ‘Io B10.


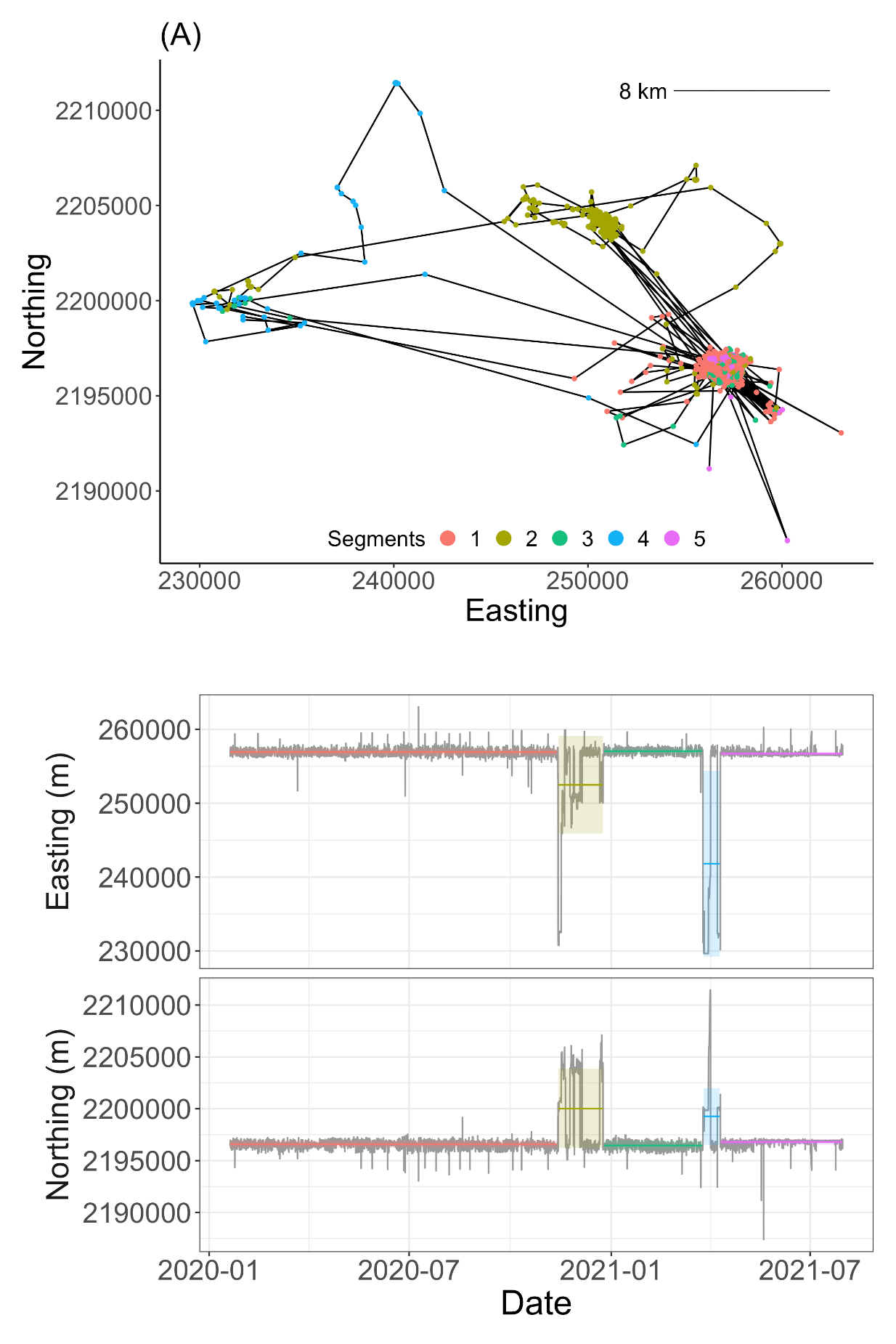


Figure S3j. Movement path (top panel) and corresponding time series of location coordinates (Easting and Northing, lower panels) for ‘Io B11.


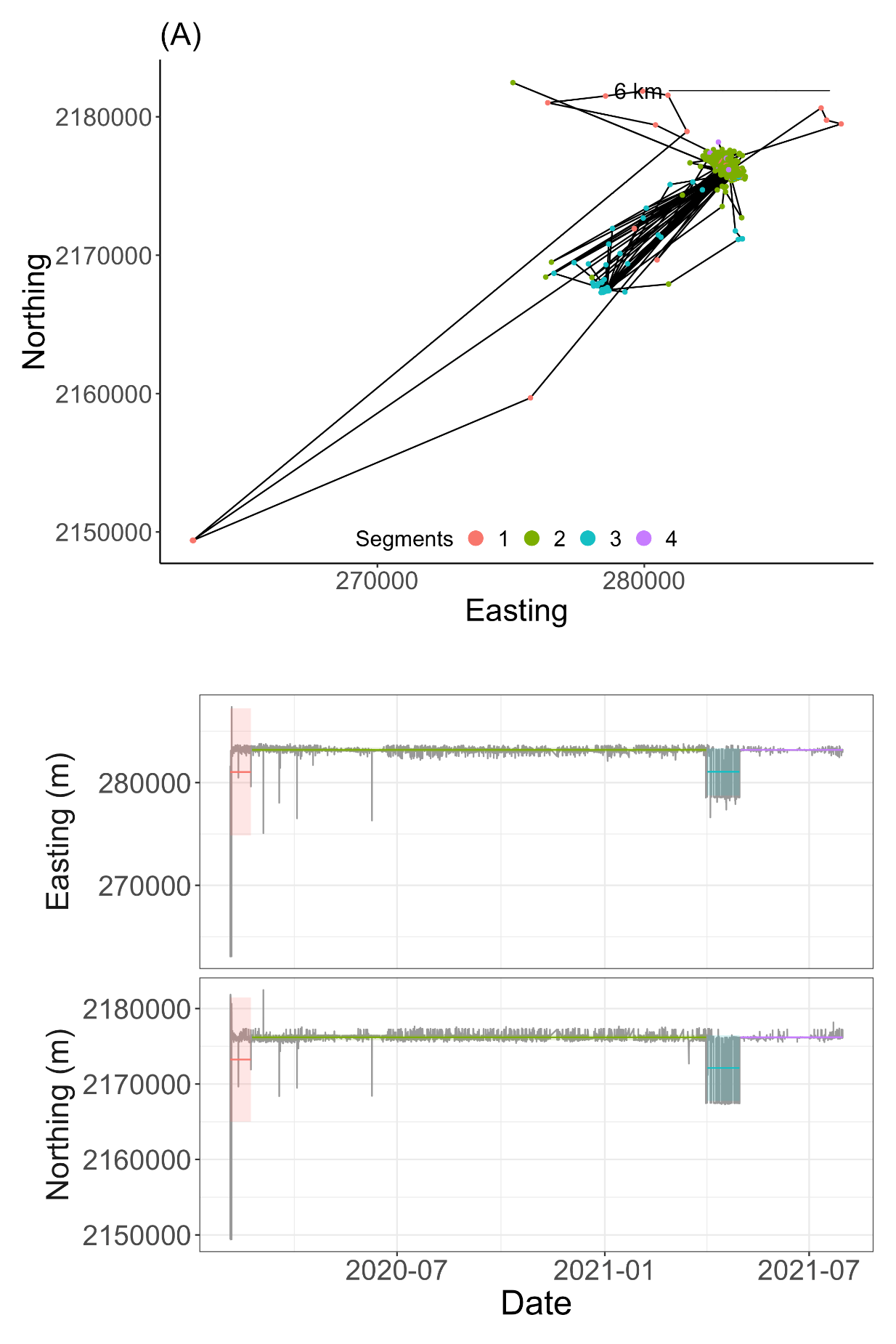


Figure S3k. Movement path (top panel) and corresponding time series of location coordinates (Easting and Northing, lower panels) for ‘Io B12.


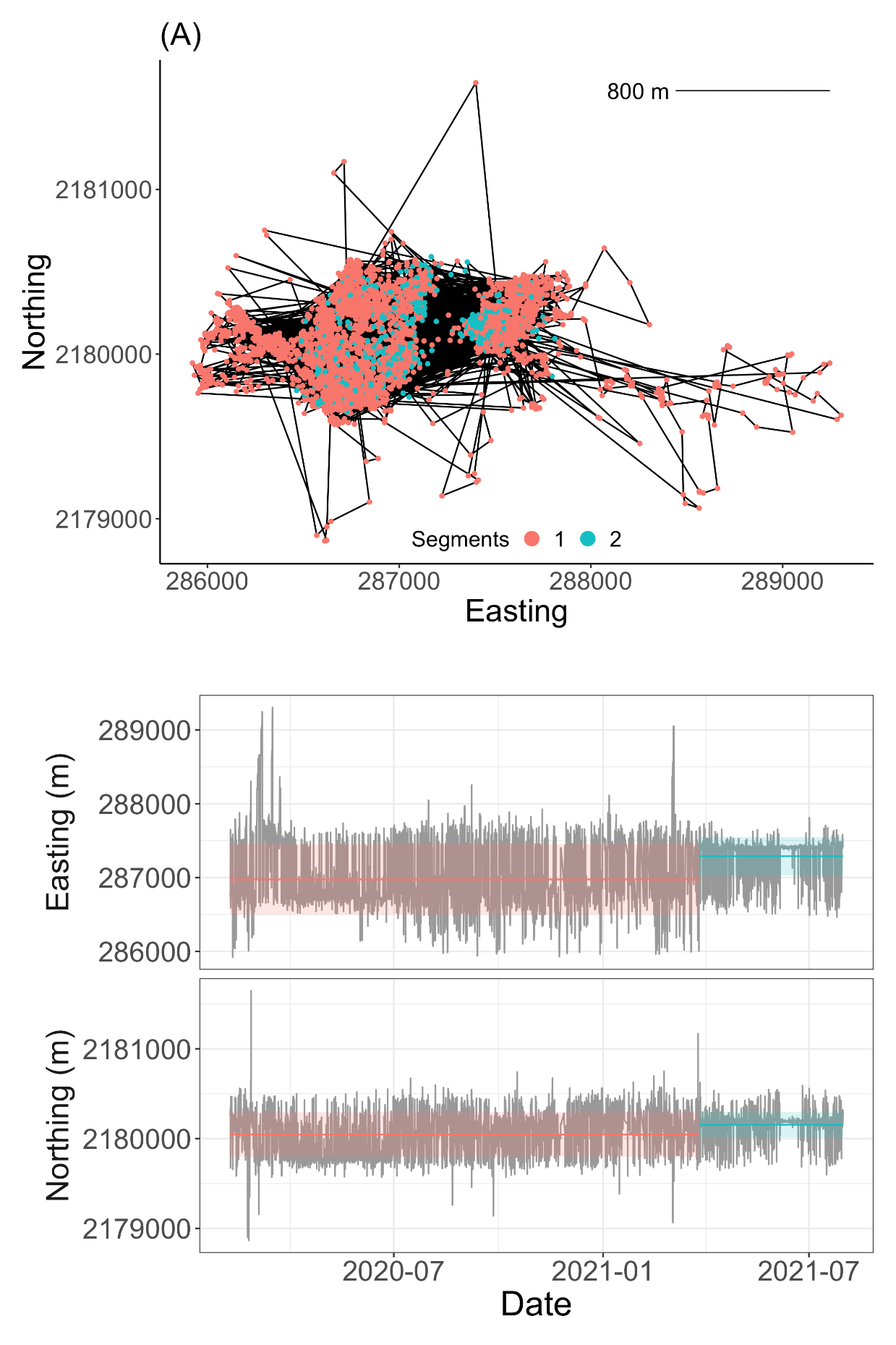


Figure S3l. Movement path (top panel) and corresponding time series of location coordinates (Easting and Northing, lower panels) for ‘Io B13.


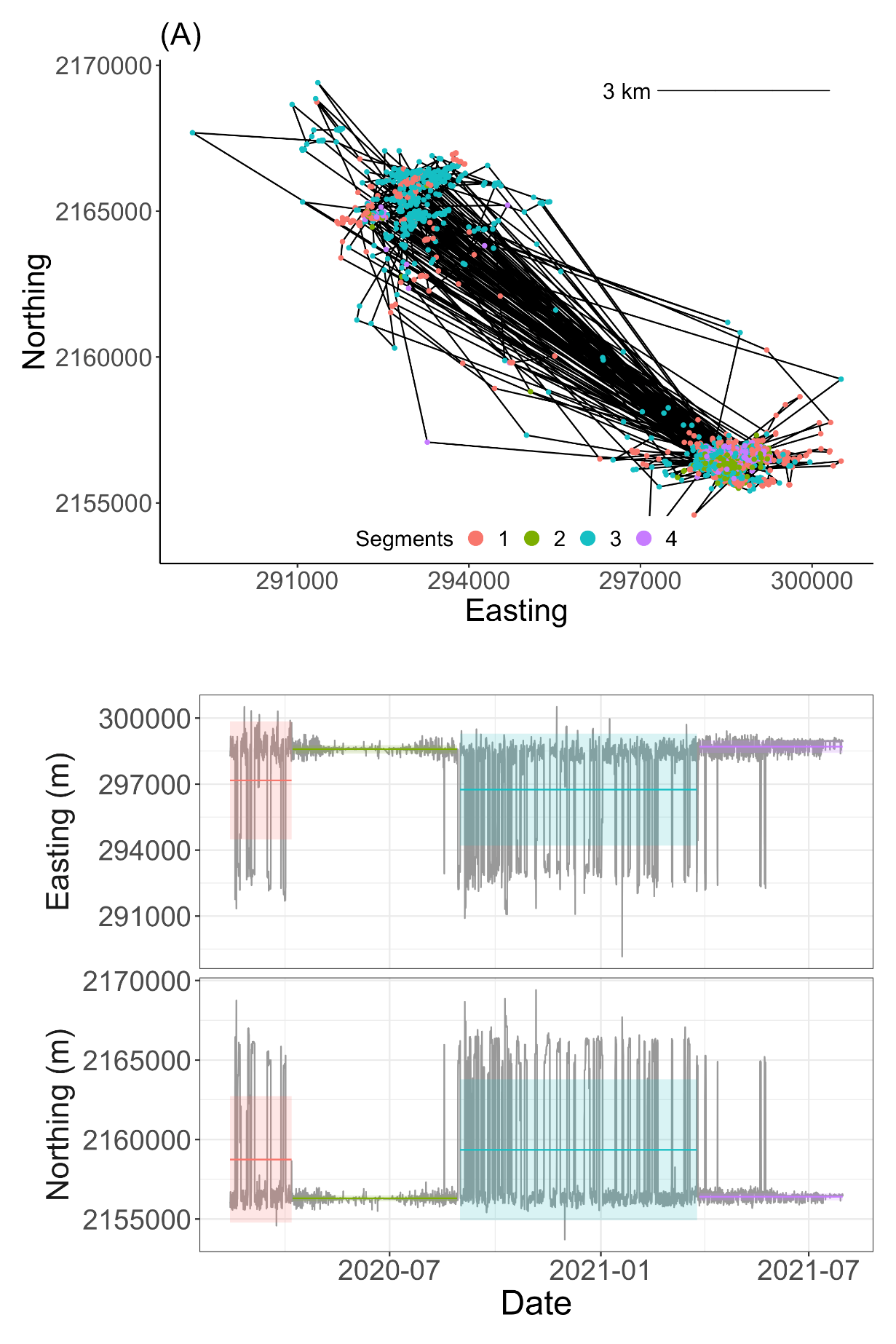


Figure S3m. Movement path (top panel) and corresponding time series of location coordinates (Easting and Northing, lower panels) for ‘Io B14.


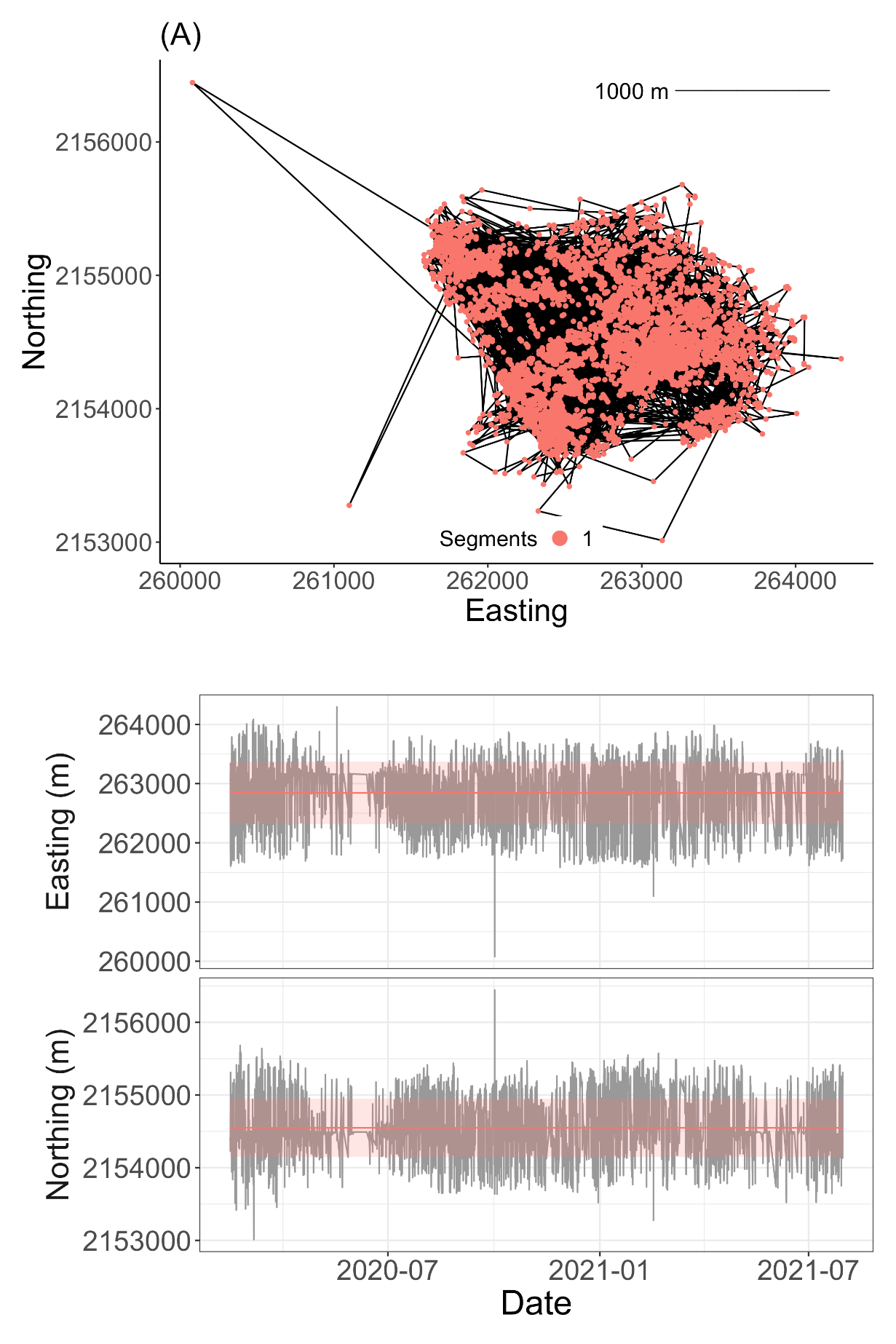


Figure S3n. Movement path (top panel) and corresponding time series of location coordinates (Easting and Northing, lower panels) for ‘Io B15.


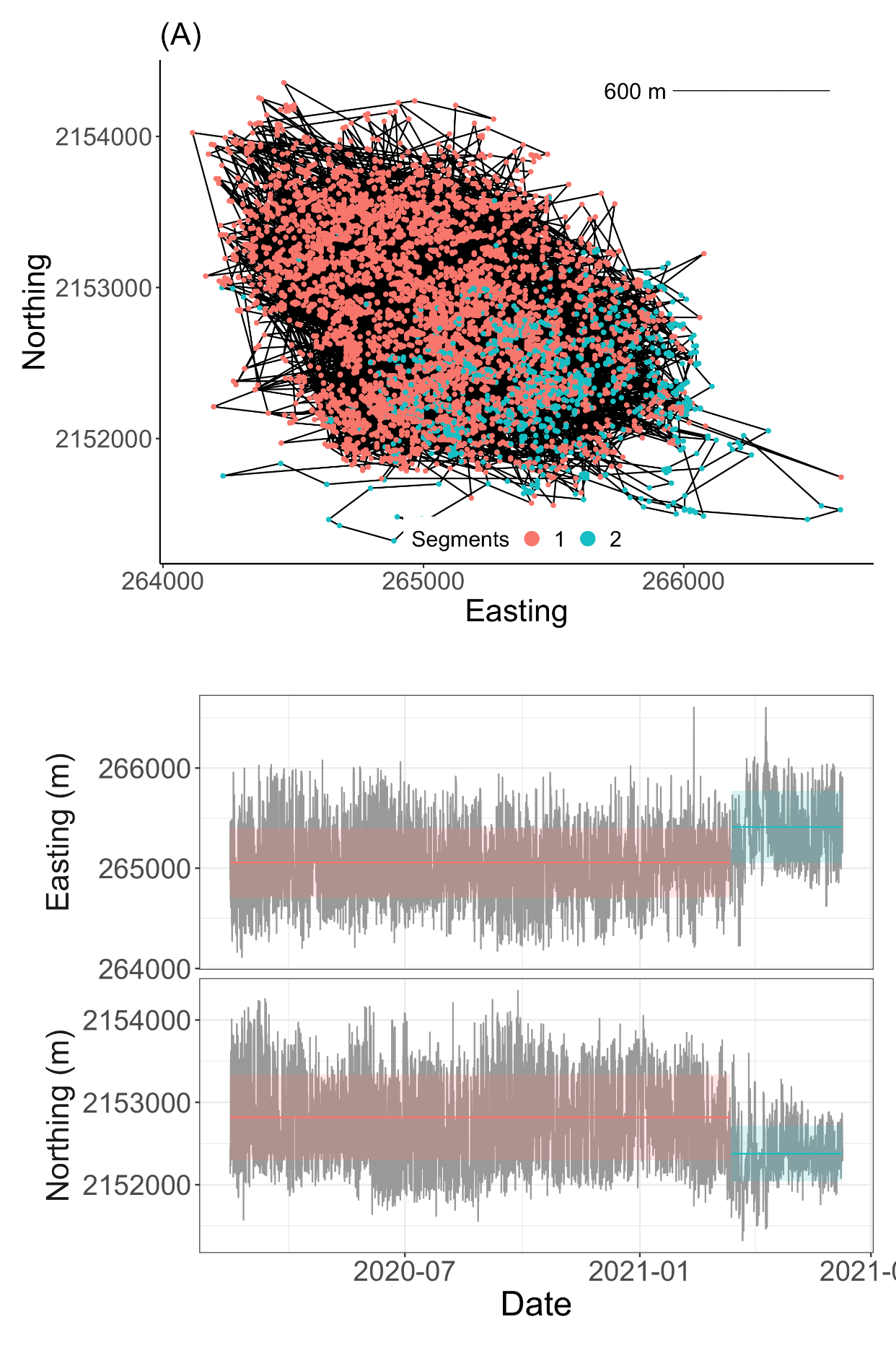


Figure S3o. Movement path (top panel) and corresponding time series of location coordinates (Easting and Northing, lower panels) for ‘Io B16.
